# Using reference intervals to improve interpretation of youth sport-related concussion biomarkers using a research platform

**DOI:** 10.1093/braincomms/fcag250

**Published:** 2026-07-05

**Authors:** Kidus Achalu, Jennifer G Cooper, Jason B Tabor, Mohammad Ghodsi, Johnny Huang, Nik Josafatow-García, Linden C Penner, Sophie Stukas, Jean-Michel Galarneau, Douglas D Fraser, Jonathan Smirl, Keith O Yeates, Chantel T Debert, Carolyn A Emery, Cheryl L Wellington

**Affiliations:** Djavad Mowafaghian Centre for Brain Health, University of British Columbia, Vancouver, British Columbia V6T 1Z3, Canada; Department of Pathology and Laboratory Medicine, University of British Columbia, Vancouver, British Columbia V6T 1Z3, Canada; Djavad Mowafaghian Centre for Brain Health, University of British Columbia, Vancouver, British Columbia V6T 1Z3, Canada; Department of Pathology and Laboratory Medicine, University of British Columbia, Vancouver, British Columbia V6T 1Z3, Canada; Faculty of Kinesiology, Sport Injury Prevention Research Centre, University of Calgary, Calgary, Alberta T2N 1N4, Canada; Hotchkiss Brain Institute, University of Calgary, Calgary, Alberta T2N 1N4, Canada; Alberta Children’s Hospital Research Institute, University of Calgary, Calgary, Alberta T2N 1N4, Canada; Djavad Mowafaghian Centre for Brain Health, University of British Columbia, Vancouver, British Columbia V6T 1Z3, Canada; Department of Pathology and Laboratory Medicine, University of British Columbia, Vancouver, British Columbia V6T 1Z3, Canada; Djavad Mowafaghian Centre for Brain Health, University of British Columbia, Vancouver, British Columbia V6T 1Z3, Canada; Department of Pathology and Laboratory Medicine, University of British Columbia, Vancouver, British Columbia V6T 1Z3, Canada; Faculty of Kinesiology, Sport Injury Prevention Research Centre, University of Calgary, Calgary, Alberta T2N 1N4, Canada; Hotchkiss Brain Institute, University of Calgary, Calgary, Alberta T2N 1N4, Canada; Alberta Children’s Hospital Research Institute, University of Calgary, Calgary, Alberta T2N 1N4, Canada; Faculty of Kinesiology, Sport Injury Prevention Research Centre, University of Calgary, Calgary, Alberta T2N 1N4, Canada; Hotchkiss Brain Institute, University of Calgary, Calgary, Alberta T2N 1N4, Canada; Alberta Children’s Hospital Research Institute, University of Calgary, Calgary, Alberta T2N 1N4, Canada; Djavad Mowafaghian Centre for Brain Health, University of British Columbia, Vancouver, British Columbia V6T 1Z3, Canada; Department of Pathology and Laboratory Medicine, University of British Columbia, Vancouver, British Columbia V6T 1Z3, Canada; Faculty of Kinesiology, Sport Injury Prevention Research Centre, University of Calgary, Calgary, Alberta T2N 1N4, Canada; Department of Pediatrics and Clinical Neurological Sciences, Western University, London, Ontario N6A 3K7, Canada; Faculty of Kinesiology, Sport Injury Prevention Research Centre, University of Calgary, Calgary, Alberta T2N 1N4, Canada; Hotchkiss Brain Institute, University of Calgary, Calgary, Alberta T2N 1N4, Canada; Alberta Children’s Hospital Research Institute, University of Calgary, Calgary, Alberta T2N 1N4, Canada; Hotchkiss Brain Institute, University of Calgary, Calgary, Alberta T2N 1N4, Canada; Alberta Children’s Hospital Research Institute, University of Calgary, Calgary, Alberta T2N 1N4, Canada; Department of Psychology, University of Calgary, Calgary, Alberta T2N 1N4, Canada; Faculty of Kinesiology, Sport Injury Prevention Research Centre, University of Calgary, Calgary, Alberta T2N 1N4, Canada; Hotchkiss Brain Institute, University of Calgary, Calgary, Alberta T2N 1N4, Canada; Alberta Children’s Hospital Research Institute, University of Calgary, Calgary, Alberta T2N 1N4, Canada; Department of Clinical Neurosciences, Cumming School of Medicine, University of Calgary, Calgary, Alberta T2N 1N4, Canada; Faculty of Kinesiology, Sport Injury Prevention Research Centre, University of Calgary, Calgary, Alberta T2N 1N4, Canada; Hotchkiss Brain Institute, University of Calgary, Calgary, Alberta T2N 1N4, Canada; Alberta Children’s Hospital Research Institute, University of Calgary, Calgary, Alberta T2N 1N4, Canada; Departments of Pediatrics and Community Health Sciences, Cumming School of Medicine, University of Calgary, Calgary, Alberta T2N 1N4, Canada; Djavad Mowafaghian Centre for Brain Health, University of British Columbia, Vancouver, British Columbia V6T 1Z3, Canada; Department of Pathology and Laboratory Medicine, University of British Columbia, Vancouver, British Columbia V6T 1Z3, Canada; School of Biomedical Engineering, University of British Columbia, Vancouver, British Columbia V6T 1Z3, Canada; International Collaboration on Repair Discoveries, University of British Columbia, Vancouver, British Columbia V6T 1Z3, Canada

**Keywords:** blood-based biomarkers, concussion, context of use, normative data

## Abstract

Youth sports-related concussion (SRC) is a major health concern. Approximately 67% of Canadian youth participate in sports and recreation, and 93% of head injuries experienced in sports and recreation are concussions. SRCs are challenging to diagnose because symptoms are often subtle, which can affect the reliability of clinical diagnostic methods that are often subjective. As undiagnosed concussions can potentially lead to unfavourable neurological implications, objective diagnostic tools have the potential to improve diagnosis and inform prognosis. Recent research has highlighted the potential utility of plasma neurofilament light (NfL) and glial fibrillary acidic protein (GFAP) for SRC diagnosis, offering a quantitative and cost-effective diagnostic method. However, a major gap in the literature is understanding whether the magnitude of change in NfL and GFAP levels post-SRC exceeds normal variation observed at the population level. This study evaluates NfL and GFAP as diagnostic SRC biomarkers by comparing preseason and post-SRC levels measured up to 28 days after injury to population-based reference intervals. Preseason (uninjured) and post-SRC plasma samples from the Surveillance in High Schools and Community Sports to Reduce Concussions and their Consequences study were quantified with Quanterix’s Neurology-4-plex-B assay and compared to previously generated age-matched reference interval. A total of 658 preseason specimens (440 male [67%]; median [range] age, 15.9 [10.4–18.4] years) and 134 post-SRC specimens (72 male [54%]; median [range] age, 16.1 [11.2–18.4] years) were assessed. Median biomarker levels were higher at post-SRC than preseason for both NfL (preseason/post-SRC = 5.1/5.5 pg/ml, *P* = 0.0339) and GFAP (preseason/post-SRC=51.7/59.7 pg/ml, *P* < 0.0001). However, when compared to population controls across the same age range, 580 (88%) preseason and 119 (89%) of post-SRC measures fell within the reference interval (5th–95th percentile) for NfL and 644 (98%) preseason and 132 (99%) of post-SRC measures fell within the reference interval for GFAP. Further, the number of participants within or above the reference interval (>95th percentile) did not differ between preseason and post-SRC groups for either NfL (*P* > 0.9999) or GFAP (*P* > 0.9999). Body mass index was inversely associated with the distribution of NfL (5th–50th/50th–95th = 23.02/22.15, *P* = 0.0083; 5th–50th/50th–95th = 24.37/21.74, *P* = 0.0038) and GFAP (5th–50th/50th–95th = 23.12/22.29, *P* = 0.0219; 5th–50th/50th–95th = 25.03/22.27, *P* = 0.0025) within the reference interval, which may explain the skew of NfL and GFAP levels in the SHRed participants compared to population controls at both preseason and post-SRC. We conclude that plasma NfL and GFAP measured up to 28 days post-SRC do not meet criteria for clinical significance as stand-alone post-acute diagnostic biomarkers for adolescent SRC.

## Teaser

Youth sport-related concussion is a major problem. Neurofilament light and glial fibrillary acidic protein are potential diagnostic biomarkers of concussion. Achalu *et al.* demonstrated that, in most youth, changes in neurofilament light and glial fibrillary acidic protein following sports-related concussion would not be classified as abnormal when compared to population-based reference intervals.

## Graphical Abstract

**Graphical Abstract:**
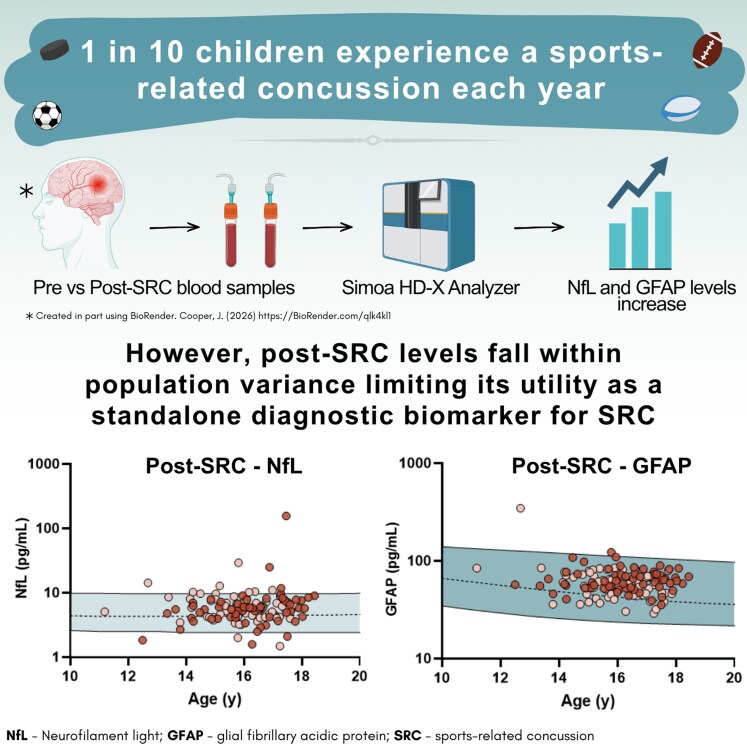

## Introduction

Sports-related concussion (SRC) is defined as an impulsive force resulting in functional and physiological changes in the brain.^1^ In Canada, ∼200 000 concussions are reported annually with the highest prevalence in adolescents.^2,3^ The gold-standard side-line assessment, the Sport Concussion Assessment Tool (SCAT), is limited by self-report subjectivity and knowledge of pre-injury status.^4-6^ Failure to diagnose a SRC can lead to premature return-to-play (RTP), persisting post-concussion symptoms and potentially serious neurological consequences.^4^ The need for reliable diagnostic and prognostic tools for SRC is high.

Blood-based proteins have potential as objective, minimally invasive and cost-effective biomarkers,^7^ with neurofilament light (NfL) and glial fibrillary acidic protein (GFAP) of interest as potential diagnostic tools for SRC.^8-13^ GFAP is an astrocyte intermediate filament protein that becomes elevated under neuroinflammatory conditions.^14^ GFAP is included in tests approved by the U.S. Food and Drug Administration (FDA) to assess the need for head CT after mild traumatic brain injury (TBI) in adults.^15^ NfL is a sensitive marker of axonal damage that becomes elevated under a broad array of neurological conditions where axonal injury is present.^16^ The Siemens Healthineers NfL assay is CE mark approved as an aid in identifying adult patients with relapsing multiple sclerosis.^17^

In most SRC studies, GFAP and NfL levels are compared between control and injury groups with significant group differences interpreted as evidence of potential diagnostic utility.^8,11,18^ The type of control group used in SRC studies varies according to study design and hypothesis tested, with data from baseline assessments, uninjured participants or those with musculoskeletal injuries being common approaches. Importantly, advancing a laboratory test towards clinical implementation also requires comparison to population norms derived from valid sampling methodology.^19^ Reference intervals (RIs) capture population variability by providing a range that encompasses the majority of normative values, enabling clinicians to interpret a test as normal or abnormal.^20^ Recently, we used plasma specimens from the Canadian Health Measures Survey (CHMS), a nationally representative survey of the Canadian population, to generate NfL and GFAP RIs that serve as population controls defining the 5th, 50th and 95th percentiles from ages 3 to 79 years.^21^

In this study, we leveraged previously published CHMS RI data^21^ extracted across ages 10–20 years to contextualize interpretation of preseason and post-SRC NfL and GFAP data collected in the Surveillance in High Schools to Reduce Concussions and Consequences of Concussions (SHRed) study. SHRed is a Canada-wide prospective longitudinal cohort study designed to investigate diagnostic, prognostic and prevention strategies for youth SRC. In the present study, we compared independent preseason and post-SRC NfL and GFAP concentrations to their respective age-appropriate RIs to determine whether adolescent SRC elevates NfL and GFAP levels beyond population-based variance.

## Materials and methods

### Ethics

The SHRed Concussions study was approved by the University of Calgary (REB18-2107) and the University of British Columbia (H19-00037) research ethics boards. Written consent was obtained from participants ≥14 years old for *n* = 738 participants (mature minor consent), and parental consent with adolescent assent was obtained for *n* = 54 participants <14 years old. Previously generated RI for plasma NfL and GFAP using banked specimens obtained from CHMS was approved by the University of British Columbia Clinical Research Ethics Board (H19-01445). The parent CHMS study was reviewed and approved by the Health Canada and the Public Health Agency of Canada Research Ethics Board, Ottawa, Canada.

### Participants and plasma specimen collection

#### SHRed concussions

SHRed Concussions (Surveillance in High School and Community Sport to Reduce Concussions and their Consequences) is a national prospective cohort study enrolling adolescents aged 10–18 years who participate in 14 high concussion risk sports (e.g. artistic swimming, baseball, basketball, cheerleading, field hockey, football, hockey, lacrosse, physical education class, ringette, ruby, soccer, volleyball, wrestling, others). Exclusion criteria encompass conditions affecting participation in sport (e.g. systemic disease, bone fractures, surgeries).

At the preseason assessment, all consenting participants completed the collection of demographic and medical history information (e.g. sex, gender, age, sport-specific years of participation, concussion history, etc.) using a Preseason Baseline Questionnaire and a preseason SCAT5 assessment that were administered by trained research staff. Preseason assessments were conducted during scheduled team practice sessions or by appointment at a research site.

SRC was diagnosed by a physician using criteria from the Berlin 2016 5th Consensus Statement on Concussion in Sport, which defines SRCs as a TBI induced by biomechanical forces that causes short-lived neurological impairment that manifests as a range of clinical signs and symptoms and largely reflects a functional disturbance rather than a structural injury.^22^ Participants who experienced an SRC underwent post-SRC SCAT5 assessments after the SRC and at associated follow-ups. Participants with clinical red flags (neck pain or tenderness, double vision, weakness or tingling/burning in arms or legs, severe or increasing headache, seizure or convulsion, loss of consciousness, deteriorating conscious state, vomiting, or increasingly restless, agitated or combative), as determined by an athletic therapist or physiotherapist, were referred to the emergency room (ER) and those confirmed with a more serious injuries (e.g. severe forms of TBI, abnormal focal neurological sign, external acute/medical conditions) were then excluded from the SHRed study and no further data were collected on these participants. SRC follow-up visits were planned for ≤72 h, 1-week post-injury and every 2 weeks thereafter until medically cleared to RTP. Clinical assessments, questionnaires and SCAT5 evaluations were completed at each follow-up.

At preseason and each post-SRC visit, ethylenediaminetetraacetic acid (EDTA) blood was collected into K2-EDTA tubes, centrifuged, aliquoted and frozen at −80°C within 2 h. Preseason blood collections were conducted either during scheduled team practice sessions or by appointment at a research site. Blood collection was not standardized for time of day or days post-SRC. Participants who had paired preseason and post-SRC specimens had their preseason specimen excluded to increase the sample size in the post-SRC group. However, an exploratory subgroup analysis was conducted using the paired samples. This secondary analysis used preseason and the first post-SRC specimen collected within 28 days, binned into 0–3, 4–10 and 11–28 days post-SRC consistent with previous SHRed studies^12^ to assess temporal effects. Additional methodological details of the SHRed Concussions study are provided in Supplemental Methods.

#### Canadian Health Measures Survey (CHMS) and RI generation

NfL and GFAP RIs were previously generated in a secondary analysis study^21^ using *n* = 900 banked plasma samples from the Statistic Canada’s Canadian Health Measures Survey (CHMS).^23^ CHMS is a population-based national survey that collects information on the health of Canadians using a representative sampling approach.^23^ The CHMS study excludes individuals who live in the three Canadian territories (Northwest Territories, Yukon and Nunavut), live on reserves and other Indigenous settlements in Canadian provinces, are full-time members of the Canadian Forces, are institutionalized, or are residents of remote regions with under 10 000 people in a 75 km radius, which together represent ∼4% of the targeted Canadian population.^23^ No further exclusion criteria were applied beyond CHMS eligibility criteria. For CHMS participants, blood was collected in EDTA vacutainers by CHMS personnel and centrifuged at 8°C for 15 min at 1800 *g*-force. Plasma was separated and banked at −80°C within 4 h of collection.

RIs for NfL and GFAP were previously generated with an even distribution of males and females (*n* = 450) selected by a Statistics Canada methodologist.^21^ RIs were generated using the Quanterix single-molecule array (Simoa) HD-X (Billerica, MA), a research-use-only platform, using the Neurology 4-Plex E (N4PE) advantage assay. RIs were generated in accordance with Clinical & Laboratory Standards Institute EP28-A3c guidelines with oversight from a Statistics Canada methodologist, a full description of RI generation can be found in Supplemental Methods. Continuous RIs were created for each analyte with the quantregGrowth package in R.^24,25^ Smoothed regression curves were generated at the 95th and 5th percentiles to represent the upper and lower limits of the RIs, respectively. A regression curve was also generated at the 50th percentile to indicate the population median. Point intervals for each age from 3 to 79 years were then created using the predict function.

For the present study, continuous RI data across the age 10–20 years were first extracted from the complete RI curves using CHMS data across the full age range of 3–79 years^21^ and used to contextualize preseason and post-SRC NfL and GFAP levels from SHRed Concussions participants who were all between ages 10 and 20 years. The full breakdown of CHMS sample sizes per age and sex bins can be found in the original RI publication.^21^ In the age bins most relevant to this study, there were *n* = 95 participants age 10–15 (*n* = 48 males, *n* = 47 females), and *n* = 57 participants age 15–20 (*n* = 32 males, *n* = 25 females).

### Biomarker analysis, cross-lot and RI harmonization

Plasma NfL and GFAP concentrations from SHRed participants were quantified using Simoa immunoassays on the Quanterix HD-X platform using the Neurology-4-plex B (N4PB) advantage assay (catalogue #103670). Each assay included an 8-point calibrator curve, two internal kit controls and three plasma controls. Specimens were diluted 4-fold on-board, assayed in duplicate, and the mean value was reported as the result. Specimens were randomized into 21 runs, keeping participants with multiple specimens on the same run. Quality was documented across all runs. For plasma controls analysed in duplicate across every plate, mean values had average inter-plate CVs between 8% and 17%. Average intra-plate CVs were between 6% and 9%. Two N4PB lots were used: lot#503228 for 149 preseason specimens collected up to November 2021 and lot#503475 for subsequent specimens.

To determine lot-to-lot bias, *n* = 35 samples from N4PB lot# 503228 were selected to be run on N4PB lot# 503475. Bland–Altman analysis was utilized to assess bias and agreement between the two N4PB lots. As the N4PE advantage assay was used to generate NfL and GFAP RIs, whereas the N4PB assay was used in the SHRed cohort, *n* = 40 samples from the SHRed cohort quantified on the N4PB assay were rerun on the N4PE advantage assay. A Weighted-Demings regression was used to transform N4PB values into an N4PE equivalent. Bland-Altman analysis was used to assess bias before and after transformation.

### Statistical analyses

Group comparisons between preseason and post-SRC for demographics, study measures, and raw biomarker concentrations were performed using a Mann–Whitney U-test for continuous variables and a Fisher’s exact test for categorical variables after testing for normality. To assess associations between demographic variables and biomarker concentrations, simple linear regression with log-transformed biomarker levels was used. To assess the association between biomarker concentrations and time point (preseason or post-SRC) while accounting for demographic variables, multivariable linear regression was performed. Regression models utilized log-transformed data to improve the normality of residuals. For analyses using sex as a variable, *n* = 1 participant with undisclosed sex was excluded. For analysis using body mass index (BMI) as a variable, *n* = 107 participants with incomplete data were excluded. For analysis using SCAT5 measures, individuals with missing data were excluded from the analysis. In laboratory medicine, it is customary to use the 97.5th and 2.5th percentiles to demarcate normal values,^26^ with 5% of the population falling outside the RI. This RI uses the 5th and 95th percentiles to reduce skewing caused by outliers or low sample numbers at either extreme.^27^ To compare the distribution of biomarker concentrations within (5th–95th percentile) and outside (>95th percentile) the RI at preseason and post-SRC, data points from SHRed samples were compared to the age-specific percentiles to determine which bin of the RI each participant’s value was (i.e. above 95th percentile, between 5th to 95th percentile). A Fisher’s exact test was then performed to compare the proportions inside and outside the RI. To compare the observed versus expected proportion of individuals in each RI section, Mee’s proportion differences and associated 95% confidence intervals (CI) were calculated and converted into a per cent difference using^28^


(1) $$Percent\;difference=\left(\frac{proportion\;difference}{expected\;proportion}\right)\times 100\;$$

To evaluate associations between clinical measures and location within the RI, continuous variables were analysed using a Mann–Whitney U-test and categorical variables used a Fisher’s exact test. All statistical analysis were two-tailed and with an *α*-level <0.05. Statistical analyses were performed using GraphPad Prism version 10.2.3 and RStudio version 4.3.1.

## Results

### SHRed cohort

**Figure 1:**
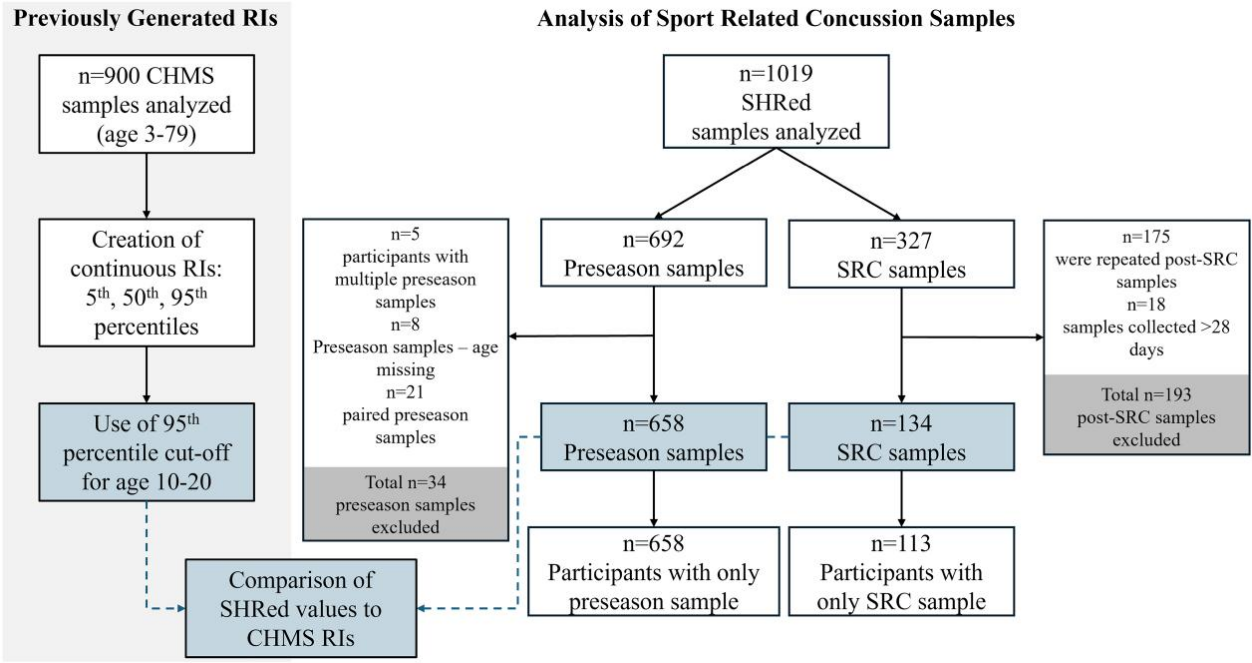
**Flow chart for plasma specimen inclusion.** Only one preseason and one post-SRC sample was included per participant. For participants with multiple preseason samples, the earliest time point in the study was selected. For participants with multiple post-SRC samples, the closest time point to concussion (most acute) was selected. Plasma specimen workflow selection for RI generation is also included.

A total of 1019 plasma specimens were collected from 807 SHRed participants (692 preseason, 327 post-SRC). The exclusion flow chart is shown in Figure 1. After exclusion, 792 specimens (658 preseason, 134 post-SRC) were included. Mann–Whitney U-test analysis revealed no significant difference in age (preseason/post-SRC = 15.9/16.1, *P* = 0.0609) or body mass index (BMI) (preseason/post-SRC = 22.4/22.5, *P* = 0.7225) between preseason and post-SRC participants; however, there were significantly more males (*P* = 0.0040) at preseason and females post-SRC (Table 1). Although this skew aligns with research indicating that females are more likely to experience an SRC,^29^ blood collection was not a mandatory component of the overall SHRed study. Thus, different consent ratios between male and female SHRed participants with respect to having preseason and post-SRC blood samples collected may have also contributed to the overall sex ratios observed here.

**Table 1:** Demographics and study measures of SHRed participants with preseason or post-SRC plasma specimens

	Preseason	Post-SRC	*P*-value
**Demographics**			
*N* (%)	658 (83)	134 (17)	
*N* (%, Post-SRC group)		35 (26, 1–3 days)	
		65 (49, 4–10 days)	
		34 (25, 11–28 days)	
Age, years (median, range)	15.9 (10.4–18.4)	16.1 (11.2–18.4)	0.0609
10–15 years old	360	62	
16–18 years old	298	72	
Male sex (*N*, %)^a^	440	72	0.0040
Female sex (*N*, %)^a^	217	62	
BMI (median, IQR)	22.4 (20.5–25.6)	22.5 (20.5–25.4)	0.7225
Referred to the ER (*n*)		16	
Days since SRC (median, IQR)		6 (3–11)	
Days to return-to-play (RTP) (median, IQR)		22 (15.0–41.3)	
Sport participation (*n*)			
• Artistic swimming	16		
• Baseball		1	
• Basketball	5	7	
• Cheerleading	1		
• Field hockey	20	1	
• Football	295	43	
• Hockey	52	8	
• Lacrosse	2	1	
• Physical education class		2	
• Ringette	15	3	
• Rugby	199	43	
• Soccer	23	9	
• Volleyball	28	7	
• Wrestling	1	1	
• Other		8	
**Study measures**			
Had previous concussion—Yes (No, %)	217 (426, 33)	65 (69, 49)	0.0015
Number of previous concussions (median, range)	0.0 (0.0–5.0)	0.0 (0.0–7.0)	0.0010
SCAT5 total symptoms (median, IQR)	5.0 (1.0–10.0)	12.0 (6.3–18.0)	<0.0001
SCAT5 symptom severity score (median, IQR)	6.0 (2.0–15.0)	22.0 (8.3–42.5)	<0.0001
NfL pg/ml (median, IQR)	5.1 (3.9–6.8)	5.5 (4.2–7.2)	0.0338
GFAP pg/ml (median, IQR)	51.7 (43.5–61.9)	59.7 (51.3–71.3)	<0.0001

Comparisons were performed using a Mann–Whitney test for continuous variables and a Fisher’s exact test for categorical variables.

^a^ *n* = 2 participants did not disclose sex.

As expected, post-SRC participants reported greater SCAT5 concussion symptom number (CSN) (preseason/post-SRC = 5/12, *P* < 0.0001) and greater SCAT5 symptom severity score (SSS) (preseason/post-SRC = 6/22, *P* < 0.0001). Also, as expected, post-SRC was associated with higher NfL levels (preseason/post-SRC = 5.1/5.5, *P* = 0.0338), and higher GFAP levels (preseason/post-SRC = 51.7/59.7, *P* < 0.0001) compared to preseason (Table 1). Differences between participants at preseason and post-SRC were observed in the proportion of individuals with a previous concussion (*P* = 0.0015) and the number of previous concussions (preseason/post-SRC=0/0, *P* = 0.0010) (Table 1). Although both preseason and post-SRC groups had medians of 0, the post-SRC group had a greater proportion of individuals with non-zero values, used for remaining analysis, yielding statistical significance.

### Cross-lot and harmonization to CHMS RI

Cross-lot and RI harmonization steps (detailed in Supplementary material) were performed following CLSI guideline EP09-A3.^30^ Bland–Altman analysis across two N4PB lots (#503228 versus #503475) showed an acceptable <15% bias for both analytes (Supplementary Fig. 1); therefore, no data correction was conducted. Cross-formulation comparison of the Quanterix N4PE to the Quanterix N4PB assay revealed a bias of 22% (SD = 15) for NfL and 28% (SD = 31) for GFAP upon Bland–Altman analysis (Supplementary Fig. 2). As bias was >20% for each analyte, a Weighted Demming regression was used to convert N4PB data to a N4PE equivalent (Supplementary Fig. 3). After correction, bias was 0.4% (SD = 15) for NfL and −0.9% (SD = 26) for GFAP (Supplementary Figs 2 and 4).

### Preseason and post-SRC biomarkers

Although significant differences were observed when comparing independent preseason and post-SRC groups, the magnitude of change was very small (Table 2). Further, a sub-analysis of paired preseason and post-SRC samples found no significant difference in biomarker levels when measured from the same participant (Table 2). Bivariate regression found that NfL was associated with age (*β* = 0.0151, *P* = 0.0379), BMI (*β* = −0.0115, *P* < 0.0001), time point (*β* = 0.0481, *P* = 0.0483) and days post-SRC (*β* = 0.0052, *P* = 0.0323) but not with sex (*β* = −0.0030, *P* = 0.8776) and collision sport (yes/no) (*β* = −0.04046, *P* = 0.0927). GFAP was directly associated with time point (*β* = 0.0519, *P* = 0.0005), days post-SRC (*β* = 0.0049, *P* = 0.0010) and was inversely associated with age (*β* = −0.0214, *P* < 0.0001), sex (*β* = −0.0355, *P* = 0.0024), BMI (*β* = −0.0073, *P* < 0.0001) and collision sport (yes/no) (*β* = −0.04309, *P* = 0.0034) (Supplementary Table 1). Multivariable linear regression using log-transformed data showed that NfL (*β* = −0.0025, *P* = 0.9446) and GFAP (*β* = 0.0266, *P* = 0.2282) were no longer associated with time point (preseason/post-SRC) after adjusting for age, sex, BMI, days post-SRC and collision sport (yes/no) (Supplementary Table 2, Supplementary Figs 5 and 6).

**Table 2:** Sub-analysis of paired samples for clinical variables, NfL and GFAP between preseason and post-SRC

		Preseason	Post-SRC	*P*-value
	Total *n* (%)	21	21	
No. of symptoms	*n* (%)	21	20	0.0110
	Median [IQR]	3.0 [1.0–9.0]	9.5 [5.5–20.0]	
Symptom severity	*n* (%)	21	20	0.0274
	Median [IQR]	3.0 [1.0–11.0]	15.5 [6.5–56.0]	
Orientation (SCAT5)	*n* (%)	21	20	>0.9999
	Median [IQR]	5.0 [5.0–5.0]	5.0 [5.0–5.0]	
Immediate Memory (of 30) (SCAT5)	*n* (%)	21	20	0.8760
	Median [IQR]	19.0 [18.5–22.5]	21.0 [19.0–22.8]	
Concentration (SCAT5)	n (%)	21	20	0.1712
	Median [IQR]	3.0 [3.0–4.0]	3.0 [2.0–3.8]	
Balance errors (SCAT5)	*n* (%)	21	19	0.1519
	Median [IQR]	2.0 [0.0–4.0]	3.0 [2.0–6.0]	
Delayed recall (SCAT5)	*n* (%)	20	20	0.1480
	Median [IQR]	7.0 [5.3–7.0]	5.5 [4.3–7.8]	
Previous concussion no.	*n* (%)	21	21	0.1250
	Median [IQR]	1.0 [0.5–3.0]	1.0 [0.0–2.5]	
BMI	*n* (%)	20	18	0.4375
	Median [IQR]	24.3 [20.5–28.1]	26.1 [22.5–28.2]	
NfL	*n* (%)	21	21	0.1111
	Median [IQR]	4.3 [3.8–5.7]	4.8 [4.1–6.5]	
GFAP	*n* (%)	21	21	0.3737
	Median [IQR]	56.5 [43.2–61.9]	57.0 [44.6–63.2]	

A Wilcoxon signed-rank test was used to analyse differences in clinical data at preseason and post-SRC.

### Comparing SHRed data to CHMS RI across age 10–20 years

**Figure 2:**
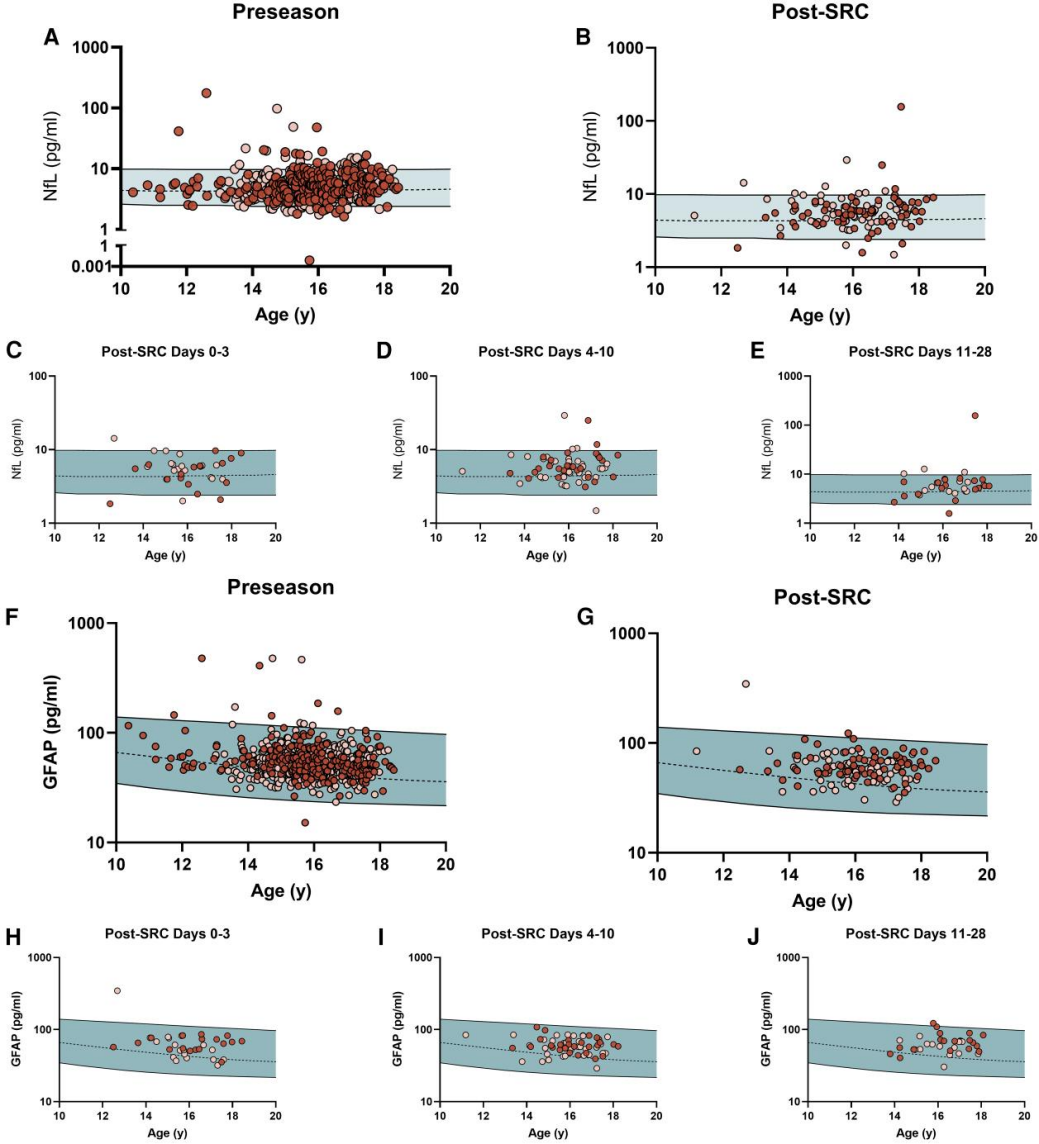
**Distribution of NfL (A–E) and GFAP (F–J) levels in participants at preseason and post-SRC.** Individual values from SHRed participants are shown in circles (preseason *n* = 658; post-SRC *n* = 134), overlaid on their respective RI (blue) where the top border represents the 95th percentile, dashed line the 50th percentile and the bottom border the 5th percentile. Colouring of dots represent participant’s sex: maroon = females, peach = male, grey = undisclosed. Panels **A**, **B**, **F** and **G** show the entire study population. Panels **C**, **D**, **E** and panels **H**, **I**, **J** show data for specific time points post-SRC for NfL and GFAP, respectively, namely Days 0–3 (**C**, **H**), 4–10 (**D**, **I**) and 11–28 (**I**, **J**).

**Figure 3:**
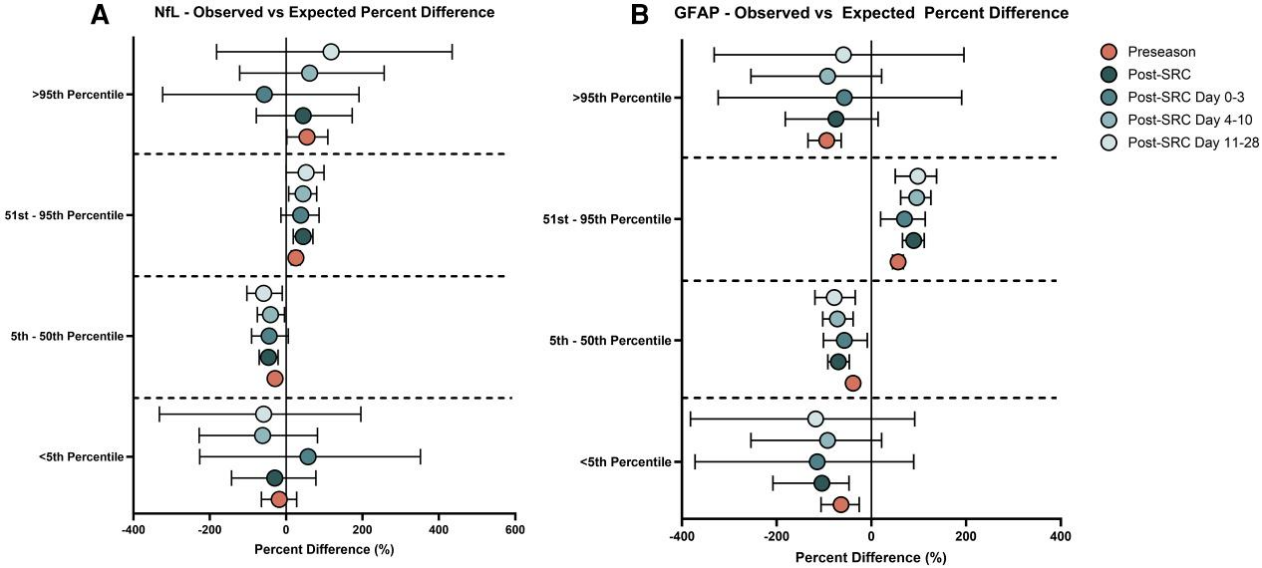
**Percent difference and its 95% confidence interval between expected and observed proportion of individuals below the 5th, between the 5th–50th and 51st–95th, and above the 95th percentile.** Graphs illustrate Mee’s proportion difference and its 95% CI for the proportion of individuals observed and expected in each section of the RI transformed into per cent differences using Eq. (1) for NfL (**A**) and GFAP (**B**). A significant difference is defined as a 95% CI that does not cross 0. Estimate and confidence intervals for post-SRC time bins are found in Supplementary Tables 3 and 4. *n* = 658 participants at preseason and *n* = 134 participants post-SRC were included.

Overlaying harmonized SHRed preseason and post-SRC NfL and GFAP levels on their respective RI from age 10 to 20 years show that most SHRed values lie within the RI, both at preseason and post-SRC, whether analysed in aggregate or when separated by days post-SRC, which were grouped into Days 0–3, 4–10 and 11–28 (Fig. 2). NfL data points had a greater than expected proportion in the 51st–95th percentile at preseason (25.3, 95% CI: 13.3–37.2) and post-SRC (44.8, 95% CI: 18.4–69.9) and less than expected in the 5th-50th percentile at preseason (−29.0, 95% CI: −40.5 to −17.4) and post-SRC (−46.4, 95% CI: −70.5 to −21.4) (Fig. 3, Table 3). Similar observations were made for GFAP, which also showed a significantly greater proportion of data points within the 51st–95th percentile at preseason (56.4, 95% CI: 44.8–67.7) and post-SRC (89.6, 95% CI: 65.6–111.5) and a lower proportion within the 5th–50th percentile at preseason (−38.5, 95% CI: −49.7 to −27.1) and post-SRC (−69.7, 95% CI: −91.8 to −46.5) (Fig. 3, Table 4). Thus, while the vast majority of preseason and post-SRC NfL and GFAP levels in SHRed participants fall within age-appropriate Canadian RIs, they are skewed towards the upper half of the RI at both preseason and post-SRC. Similar results were found when analysing the proportion of individuals in each section of the RI by time bins (Supplementary Tables 3 and 4). No significant difference was found when comparing the proportion of individuals within each section of the RI across different time bins (Supplementary Table 5). Therefore, aggregate post-SRC data was used for the remaining analysis.

**Table 3:** Expected and observed frequencies for harmonized NfL levels as well as their per cent difference and 95% CI for various section of the NfL RI.

NfL	Preseason				Post-SRC			
	Expected	Observed	Percent difference	95% Confidence interval	Expected	Observed	Percent difference	95% Confidence interval
<5th percentile	33 (5%)	27 (4%)	−18.2	(−64.9 to 27.6)	7 (5%)	5 (4%)	−29.9	(−142.9 to 78.0)
5th–50th percentile	296 (45%)	209 (32%)	−29.0	(−40.5 to −17.4)^a^	60 (45%)	32 (24%)	−46.4	(−70.5 to −21.4)^a^
51st–95th percentile	296 (45%)	371 (56%)	25.3	(13.3–37.2)^a^	60 (45%)	87 (65%)	44.8	(18.4–69.9)^a^
>95th percentile	33 (5%)	51 (8%)	54.7	(1.9–109.12)^a^	7 (5%)	10 (7%)	44.8	(−78.0 to 172.8)
<50th percentile	329 (50%)	236 (36%)	−28.3	(−38.8 to −17.6)^a^	67 (50%)	37 (28%)	−44.8	(−66.8 to −21.6)^a^
5th–95th percentile	592 (90%)	580 (88%)	−2.0	(−5.8 to 1.74)	121 (90%)	119 (89%)	−1.7	(−10.1 to 6.7)^a^
>50th percentile	329 (50%)	422 (64%)	28.3	(17.6–38.8)^a^	67 (50%)	97 (72%)	44.8	(21.6–66.8)^a^

Expected counts are the expected number of individuals in each section of the RI based on *n* = 658 preseason and *n* = 134 post-SRC specimen. Per cent differences and 95% CI were calculated using Mee’s proportion difference method and Eq. (1). A significant difference is defined as a 95% CI that does not cross 0.

^a^Represents significant results.

**Table 4:** Expected and observed frequencies for harmonized GFAP levels as well as their per cent difference and 95% confidence interval (CI) for various section of the GFAP RI

GFAP	Preseason				Post-SRC			
	Expected	Observed	Percent difference	95% Confidence interval	Expected	Observed	Percent difference	95% Confidence interval
<5th percentile	33 (5%)	2 (0%)	−94.2	(−133.7 to −63.5)^a^	7 (5%)	0 (0%)	−104.5	(−207.8 to −47.3)^a^
5th–50th percentile	296 (45%)	181 (28%)	−38.5	(−49.7 to −27.1)^a^	60 (45%)	18 (13%)	−69.7	(−91.8 to −46.5)^a^
51st–95th percentile	296 (45%)	463 (70%)	56.4	(44.8–67.7)^a^	60 (45%)	114 (85%)	89.6	(65.6–111.5)^a^
>95th percentile	33 (5%)	12 (2%)	−63.8	(−106.2 to −25.6)^a^	7 (5%)	2 (1%)	−74.6	(−181.8 to 14.2)
<50th percentile	329 (50%)	183 (28%)	−44.4	(−54.5 to −34.0)^a^	67 (50%)	18 (13%)	−73.1	(−92.9 to −52.0)^a^
5th–95th percentile	592 (90%)	644 (98%)	8.8	(6.1–11.8)^a^	121 (90%)	132 (99%)	9.1	(3.4–16.2)^a^
>50th percentile	329 (50%)	475 (72%)	44.4	(34.0–54.5)^a^	67 (50%)	116 (87%)	73.1	(52.0–92.9)^a^

Expected counts are the expected number of individuals in each section of the RI based on *n* = 658 preseason and *n* = 134 post-SRC specimen. Per cent differences and 95% CI were calculated using Mee’s proportion difference method and Eq. (1). A significant difference is defined as a 95% CI that does not cross 0.

^a^Represents significant results.

### Implications of using the 95th percentile as a cutoff point to define abnormal biomarker concentrations

For NfL, 580 (88%) preseason and 119 (89%) post-SRC measures fell within the RI (5th–95th percentile), while 51 (8%) preseason and 10 (7%) post-SRC measures were above the 95th percentile (Table 3). For GFAP, 644 (98%) preseason and 132 (99%) post-SRC measures fell within the RI and 12 (2%) preseason and 2 (1%) post-SRC measures were above the 95th percentile (Table 4). Neither biomarker showed associations between time point (preseason/post-SRC) and levels above the RI (NfL *P* > 0.9999, GFAP *P* > 0.9999) (Supplementary Table 6).

We qualitatively examined a case series of participants with post-SRC NfL or GFAP levels above the 95th percentile, whose specimens were collected a median of 10 days post-injury (Supplementary Table 7). Eight (72%) of these participants had an RTP of ≥26 days, with the longest being 11 weeks (median = 30 days). This is longer than the median RTP of 21 days for the post-SRC SHRed cohort, although it falls within the consensus recovery period of 28 days.^22^ Notably, only 43% of participants with biomarkers below the 95th percentile have RTP ≥26 days. As SHRed participants who presented with a clinical red flag were noted in the SCAT5 and referred to the ER (*n* = 16), we also examined their RTP (median = 44 days), NfL, and GFAP levels. All had biomarker levels within the RI except for one individual with NfL below the 5th percentile (Supplementary Fig. 7). Thus, biomarkers elevated above the 95th percentile could potentially predict slower recovery in the absence of clinical red flags. Notably, participants who had biomarker levels above the 95th percentile were not the same individuals who underwent ER assessment post-SRC.

### Factors associated with biomarker levels

Given that NfL and GFAP data points consistently fell within the upper half (51st–95th percentile) of the RI at preseason and post-SRC (Tables 3 and 4), we compared demographics and study measures between participants in the lower (5th–50th percentile) and upper halves of the RI for preseason and post-SRC (Supplementary Tables 8 and 9). At preseason, SCAT5 SSS (lower/upper = 5/7, 95% CI: 0.0–3.0, *P* = 0.0205) and CSN (lower/upper = 4/5, 95% CI: 0.0–2.0, *P* = 0.0066) were significantly higher for individuals in the upper half of the GFAP RI than those in the lower half. BMI was significantly lower for individuals in the upper half of the RI for NfL at preseason (lower/upper = 23.0/22.2, 95% CI: −1.6 to −0.2, *P* = 0.0083) and post-SRC (lower/upper = 24.0/21.9, 95% CI: −3.8 to −0.6, *P* = 0.0079) and for GFAP at preseason (lower/upper = 23.1/22.3, 95% CI: −1.6 to −0.1, *P* = 0.0219) and at post-SRC (lower/upper = 25.0/22.3, 95% CI: −5.0 to −1.1, *P* = 0.0025). As many SHRed participants played football and rugby, we also evaluated preseason and post-SRC NfL and GFAP levels in participants in each sport and observed significant differences for GFAP levels in rugby (*P* = 0.0015) and no difference in football (*P* = 0.0933) as well as NfL levels in rugby (*P* = 0.0891) and football (*P* = 0.5227) (Supplementary Fig. 8).

## Discussion

This study is the first to contextualize plasma NfL and GFAP concentrations in participants from the SHRed Concussions study relative to Canadian population-based RI.^21^ Using the 95th percentile as a conventional cut-off to define elevated NfL and GFAP levels revealed that neither biomarker on its own reliably associates with a clinical SRC diagnosis. Despite subtle but significant group differences between preseason and post-SRC levels, which is consistent with many previous reports,^12,31^ most individual biomarker levels at both preseason and post-SRC were within the normative range (5th–95th percentile), a finding that indicates neither NfL nor GFAP has clinically meaningful potential to serve as a standalone post-acute diagnostic biomarkers of adolescent SRC when measured at the time points assessed here.

Previous studies have suggested potential diagnostic utility of NfL and GFAP in SRC. Although many studies, including this one, report elevated NfL and GFAP following a SRC, the majority of studies have focused on adult or professional athlete populations, and many included acute blood sampling (24–48 h).^9,32-34^ Although acute sampling is very feasible for adults, the SHRed study focused on adolescent athletes with an optional consent for blood sampling, and thus acute blood collection proved challenging in this cohort. Acute sampling, especially within the first 48 h after SRC, may have revealed biomarker differences that would be interpreted as abnormal. Additionally, the majority of previous studies have focused on group-to-group comparisons, whereas this study goes beyond standard group comparisons by benchmarking preseason and post-SRC biomarker levels against a population-based RI.

To our knowledge, this is the first SRC study that considers biomarker changes in the context of valid and age-appropriate RI to better reflect biological variability of the intended target population. Both preseason and post-SRC NfL and GFAP levels in SHRed participants were skewed towards the upper half of their respective RIs, with ≥58% of data points being in the upper half (50th–95th percentile). BMI was significantly inversely associated with this skew at preseason and post-SRC for both biomarkers, with those in the upper half of the RI having significantly lower BMI. These findings align with other studies reporting an inverse relationship between NfL, GFAP and BMI,^35-37^ attributable to greater blood volume that dilutes biomarker concentrations at higher BMI.^35^ The median SHRed BMI is 22.46 kg/m^2^, similar to the 2009 average BMI in Canadian youth aged 12–19 of 22.5 kg/m^2^,^38^ whereas CHMS specimens were collected in 2016–2017 and encompass both active and sedentary individuals. Alternatively, SHRed preseason levels may skew high due to a potentially higher concussion history than the general population.

Post-SRC SCAT5 SSS and CSN were associated with the skew from the lower to the upper half of the RI for preseason GFAP levels. As GFAP reflects astrocyte inflammation,^14^ high preseason GFAP suggests potential chronic or recent neuroinflammation, the aetiology of which remains to be determined. Surprisingly, SSS and CSN were not associated with the skew between the lower and upper half of the RI for GFAP at post-SRC. While we would expect a difference in SSS and CSN between the lower and upper half of the RI post-SRC, this discrepancy is likely due to individuals in the lower half also reporting higher SSS and CSN associated with the SRC.^39^

While this study demonstrates that NfL and GFAP have limited clinical utility as standalone post-acute diagnostic biomarkers of adolescent SRC when compared to population-based RIs, they may have potential value for risk stratification. Importantly, although few SHRed post-SRC values would be classified as abnormal for either biomarker using the 95th percentile as a conventional cut-off, 8 of 11 participants with post-SRC NfL or GFAP levels falling above the 95th percentile and no clinical red flags trended towards a longer RTP than the median RTP for SHRed post-SRC cohort (Supplementary Table 10). The breakdown of RTP and other variables for post-SRC subgroups is shown in Supplementary Table 11. Further investigation is required to ascertain if the SRC recovery trajectory is associated with NfL and GFAP levels.

Future studies could also consider whether plasma NfL and GFAP may be more informative when interpreted in terms of intra-person percentile change, similar to how crossing percentiles in paediatric growth charts indicates a clinical concern.^40^ Comparison to a personal baseline is not uncommon in sports medicine, with many professional sports conducting preseason SCAT5s.^41^ Whether intra-individual percentile change from baseline may be clinically useful with these biomarkers requires further research.

Our study has several limitations. First, the sample size precluded dividing post-SRC time bins more narrowly. The median [IQR] number of days post-SRC was 6 [3–10], with the earliest and latest being 1 and 28 days after injury, respectively. Thus, peak GFAP levels, which occur within 24 h post-SRC in adults,^10,18,42^ are likely to have been missed. However, a median of 6 days post-SRC is within the expected peak of 6–10 days for NfL.^42,43^ As SRC symptoms can manifest over time, individuals vary in when they seek medical attention.^2,44^ Second, the SHRed Concussions research programme intentionally included a diverse group of sports with high concussion risk, which leads to heterogeneity of sports played in preseason versus post-SRC groups. As our intention was to determine the potential utility of NfL and GFAP to assist in the diagnosis of overall SRC concussion rather than identify sport-specific biomarkers, further research will be needed to evaluate potential cross-sport differences in biomarker responses. Third, SHRed data on exposure to head acceleration events (HAE) are not available at this time, and thus, the effect of HAE on plasma biomarkers will require future studies to assess. Fourth, CHMS RI reflect population-based norms rather than report on levels in predetermined ‘healthy’ individuals.^21^ Individuals with underlying health conditions were not excluded from CHMS and could contribute to population variance. However, population-based RIs increase generalizability because they better reflect population norms and are more representative of the target population. Fourth, the relationship between NfL, GFAP and BMI requires further investigation. BMI only considers body weight and height rather than body composition; therefore, muscular individuals may be classified as having a high BMI but may not demonstrate the same relationship.^45^ Finally, the Quanterix Simoa platform used here is research-use-only.

## Conclusion

In adolescents, plasma NfL and GFAP have limited utility as stand-alone post-acute diagnostic biomarkers for SRC when using the age-adjusted 95th percentile in population-based RIs as a cut-off to demarcate elevated biomarker levels. These results suggest that NfL and GFAP may not be useful in a clinical setting where baseline samples are not available, and RIs are thus necessary to define normal and abnormal levels in adolescent athletes. Notably, although the timing of post-SRC blood measures is undeniably important, potential biomarker changes within the first 24 h post-SRC could not be assessed in this study. Adjusting for BMI may increase the precision and accuracy of GFAP and NfL interpretation in adolescents. Future studies may show utility for NfL and GFAP as risk stratification biomarkers to identify adolescents who may experience longer than average RTP after SRC.

## Supplementary Material

fcag250_Supplementary_Data

## Data Availability

As per the agreement with the funder data are not available. Please contact Dr. Carolyn Emery for any questions about SHRed data.
