# Supplementary material for: Using reference intervals to improve interpretation of youth sport-related concussion biomarkers using a research platform
Source: Brain Commun. 2026 Jul 5;8(4):fcag250. doi: 10.1093/braincomms/fcag250 (PMC13378169; doi:10.1093/braincomms/fcag250)
Supplement: fcag250_Supplementary_Data [file fcag250_supplementary_data.pdf]

Supplemental Methods and Results

Participants and specimen collection

SHRed Concussions (Surveillance in High School and Community Sport to Reduce Concussions and their Consequences) is a national prospective cohort study enrolling adolescents aged 10-18 years who participate in 14 high concussion risk sports (e.g., artistic swimming, baseball, basketball, cheerleading, field hockey, football, hockey, lacrosse, physical education class, ringette, ruby, soccer, volleyball, wrestling, others). Exclusion criteria encompass conditions affecting participation in sport (e.g., systemic disease, bone fractures, surgeries). For all consenting participants, pre-season assessment including demographic and medical history information (e.g., sex, gender, age, sport-specific years of participation, concussion history, etc.) were collected using a Preseason Baseline Questionnaire (PBQ) and the Sport Concussion Assessment Tool 5 (SCAT5) by trained research staff. Individual participation exposure hours (e.g., practice, game) were collected on a weekly exposure sheet (WES) by a team designate. SRC diagnoses were made by a study physician using criteria outlined by the Berlin 2016 5th Consensus Statement on Concussion in Sport (full description cited below)[1]. Participants with any clinical red flag (e.g., neck pain or tenderness, double vision, weakness or tingling/burning in arms and legs, severe or increasing headache, seizure or convulsion, loss of consciousness, deteriorating conscious state, vomiting, increasingly restless, agitated or combative), were directed to the emergency room (ER) for assessment. If cleared of a more serious injury, these individuals could follow-up with a SHRed study physician after the ER visit. SRC follow-up visits were a priori planned to occur ≤72 hours, 1-week post-injury, and every 2 weeks thereafter until medically cleared to return-to-play (RTP). However, this study's secondary analysis only included specimens collected at the first SRC visit,

which occurred within 28 days after injury and were binned into 0-3, 4-10, and 11-28 days post-injury groups. Clinical assessments, questionnaires, and SCAT5 evaluations were completed at each follow-up. At preseason and post-SRC visits, venous blood was collected into K2-EDTA vacutainer tubes, centrifuged at room temperature for 10 minutes at 1300g, and plasma was aliquoted and frozen at -80°C within two hours of collection. Plasma samples were shipped on dry ice to The University of British Columbia (UBC) where they were analyzed in a central laboratory on the Quanterix Simoa HD-X platform using the Neurology-4-Plex B (N4PB) assay.

Berlin 2016 5th Consensus Statement[1]

Sport related concussion is a traumatic brain injury induced by biomechanical forces. Several common features that may be utilized in clinically defining the nature of a concussive head injury include:

- SRC may be caused either by a direct blow to the head, face, neck or elsewhere on the body with an impulsive force transmitted to the head.
- SRC typically results in the rapid onset of short-lived impairment of neurological function that resolves spontaneously. However, in some cases, signs and symptoms evolve over a number of minutes to hours.
- SRC may result in neuropathological changes, but the acute clinical signs and symptoms largely reflect a functional disturbance rather than a structural injury and, as such, no abnormality is seen on standard structural neuroimaging studies.
- SRC results in a range of clinical signs and symptoms that may or may not involve loss of consciousness. Resolution of the clinical and cognitive features typically follows a sequential course. However, in some cases symptoms may be prolonged.

The clinical signs and symptoms cannot be explained by drug, alcohol, or medication use, other injuries (such as cervical injuries, peripheral vestibular dysfunction, etc.) or other comorbidities (e.g., psychological factors or coexisting medical conditions).

SHRed Concussion Diagnosis Criteria:

SHRed participants who experienced a suspected SRC in accordance with the Concussion Recognition Tool 5 were removed from play and completed a sideline assessment using the SCAT5 in the presence of an athletic therapist or physiotherapist. Parents of SHRed participants who experienced a suspected SRC from community sports communicated with the school-based clinical coordinator or a research team member at the site and a follow-up plan was established for physician assessment. Physician diagnosis was confirmed upon:

- Identification of a reportable/suspected concussion
- Documentation of at least one sign or symptom of a possible concussion at the time of the medical assessment or between identification of a reportable concussion and medical assessment
- Absence of developing red flags as listed in the SCAT5
- Absence of the need to investigate more severe forms of TBI (e.g., CT scan rule)
    - If additional investigation is warranted, concussion diagnosis can occur after negative investigation results are obtained
- Absence of abnormal focal neurological sign upon neurological assessment
- Absence of acute condition other than concussion that can explain ALL reported symptoms

- Absence of pre-existing or developing health conditions that can explain ALL reported symptoms
- No use of drug, alcohol, or medication that can explain the clinical presentation"

Reference Interval Generation

NfL and GFAP reference intervals (RIs) were previously generated using banked plasma specimens from Statistics Canada's Canadian Health Measures Survey (CHMS)[2]. As described in this previously published study, specimens were collected by the CHMS in 2016 and 2017 from Canadians aged 3-79 years old and banked.[3] CHMS excludes approximately 4% of the Canadian population, including those who live in the three Canadian territories, on reserves or Indigenous settlements, or are full-time members of the Canadian Forces.[3] In the previously published RI study, 4 participants (2 male, 2 female) across the 16 CHMS collection sites for each age group (3-5, 6-11, 12-19, 20-39, 40-59, 50-79 years) were initially selected (n=384) to determine how many additional specimens would be needed to establish the RIs.[2] A Statistics Canada methodologist determined n=516 additional plasma specimen would be needed across age bins to establish RIs and selected these specimens to reflect an even distribution of sex and geographical sites of collection. RI generation conformed to Clinical & Laboratory Standards Institute EP28-A3c guidelines. Banked plasma NfL and GFAP levels were quantified using the single-molecule array enzyme linked immunoassay (Simoa) HD-X platform from Quanterix (Billerica, MA) using the Neurology-4-plex E (N4PE) assay (catalogue #103670, lot#503105) according to the manufacturer's protocol. Continuous RIs for NfL and GFAP were generated using the *quantregGrowth* package in the R Statistical Programming Language. Smoothed regression curves were produced at the 5th, 50th, and 95th percentiles to represent the lower limit, population median, and upper limit of the RI. 95% CI were generated for the lower and upper limit of the regression

curves. The smoothing factor (lambda) for each regression function was determined by cross validation and adjusted based on biological expectations.

Cross-lot and Reference Interval harmonization

SHRed specimens were analyzed using the Neurology-4-plex B (N4PB) assay over 5 runs between Oct to Nov 2021 (lot #503228) and 16 runs between Dec 2022 to Jan 2023 (lot #503475). As NfL and GFAP levels were measured across two different lots, cross-lot analysis was conducted by analyzing n=35 specimens originally run on N4PB lot #503228 on lot #503475. Bland-Altman analysis, which calculates (Method A – Method B)/mean*100 for all paired points and then calculates the average percent difference across all pairs of points, revealed <15% bias between lots for both analytes (**Supplementary Fig. 1**). As this is below the 20% threshold for correction, no data correction was conducted across assay lots.

The next step was to harmonize SHRed data with age-specific RIs. Previously derived RIs for NfL and GFAP from 900 CHMS specimens aged 3-79 years used the Quanterix Neurology-4-plex E (N4PE) advantage assay (catalogue #103670, lot #503105) and defined the continuous 5th, 50th, and 95th percentiles[2]. The 5th-95th percentile range was chosen to reduce skew caused by outliers and small sample size at either extreme. Because SHRed specimens were analyzed using a different assay formulation than the RIs (N4PB vs N4PE), a cross-formulation comparison was performed following CLSI guideline EP09-A3.[4] A total of n=40 SHRed specimens selected to cover the maximum dynamic range (i.e. from lowest to highest concentrations available) of NfL and GFAP were re-analyzed using N4PE assay lot 503940.[4] SHRed specimens were ordered from highest to lowest based on GFAP concentration and randomly selected with a greater number of specimens being selected from the higher and lower ends. For both analytes, concentrations strongly correlated (NfL rho=0.92, p<0.0001; GFAP rho=0.85, p<0.0001), however, Bland-

Altman analysis revealed a bias of 22% (SD=15) for NfL and 28% (SD=31) for GFAP (**Supplementary Fig. 2**). A Weighted Demming regression (**Supplementary Fig. 3**) was used to convert N4PB data to a N4PE equivalent. After correction, bias was 0.4% (SD=15) for NfL and -0.9% (SD=26) for GFAP (**Supplementary Fig. 2**). SHRed data were harmonized to the N4PE assay using regression formulas reported in **Supplementary Fig. 3**. Original and harmonized data are presented in **Supplementary Fig. 5**.

References

(1) McCrory, P.; Meeuwisse, W.; Dvorak, J.; Aubry, M.; Bailes, J.; Broglio, S.; Cantu, R. C.; Cassidy, D.; Echemendia, R. J.; Castellani, R. J.; Davis, G. A.; Ellenbogen, R.; Emery, C.; Engebretsen, L.; Feddermann-Demont, N.; Giza, C. C.; Guskiewicz, K. M.; Herring, S.; Iverson, G. L.; Johnston, K. M.; Kissick, J.; Kutcher, J.; Leddy, J. J.; Maddocks, D.; Makdissi, M.; Manley, G. T.; McCrea, M.; Meehan, W. P.; Nagahiro, S.; Patricios, J.; Putukian, M.; Schneider, K. J.; Sills, A.; Tator, C. H.; Turner, M.; Vos, P. E. Consensus Statement on Concussion in Sport—the 5th International Conference on Concussion in Sport Held in Berlin, October 2016. *Br. J. Sports Med.* **2017**, *51* (11), 838–847. https://doi.org/10.1136/bjsports-2017-097699.

(2) Cooper, J. G.; Stukas, S.; Ghodsi, M.; Ahmed, N.; Diaz-Arrastia, R.; Holmes, D. T.; Wellington, C. L. Age Specific Reference Intervals for Plasma Biomarkers of Neurodegeneration and Neurotrauma in a Canadian Population. *Clin. Biochem.* **2023**, *121–122*, 110680. https://doi.org/10.1016/j.clinbiochem.2023.110680.

(3) Government of Canada, S. C. *Canadian Health Measures Survey (CHMS)*. https://www23.statcan.gc.ca/imdb/p2SV.pl?Function=getSurvey&Id=251160 (accessed 2025-09-17).

(4) CLSI. *Measurement Procedure Comparison and Bias Estimation Using Patient Samples; Approved Guideline - Third Edition*; CLSI Guideline EPO9-A3; Clinical and Laboratory Standards Institute, 2013.

Supplementary Figures and Tables:

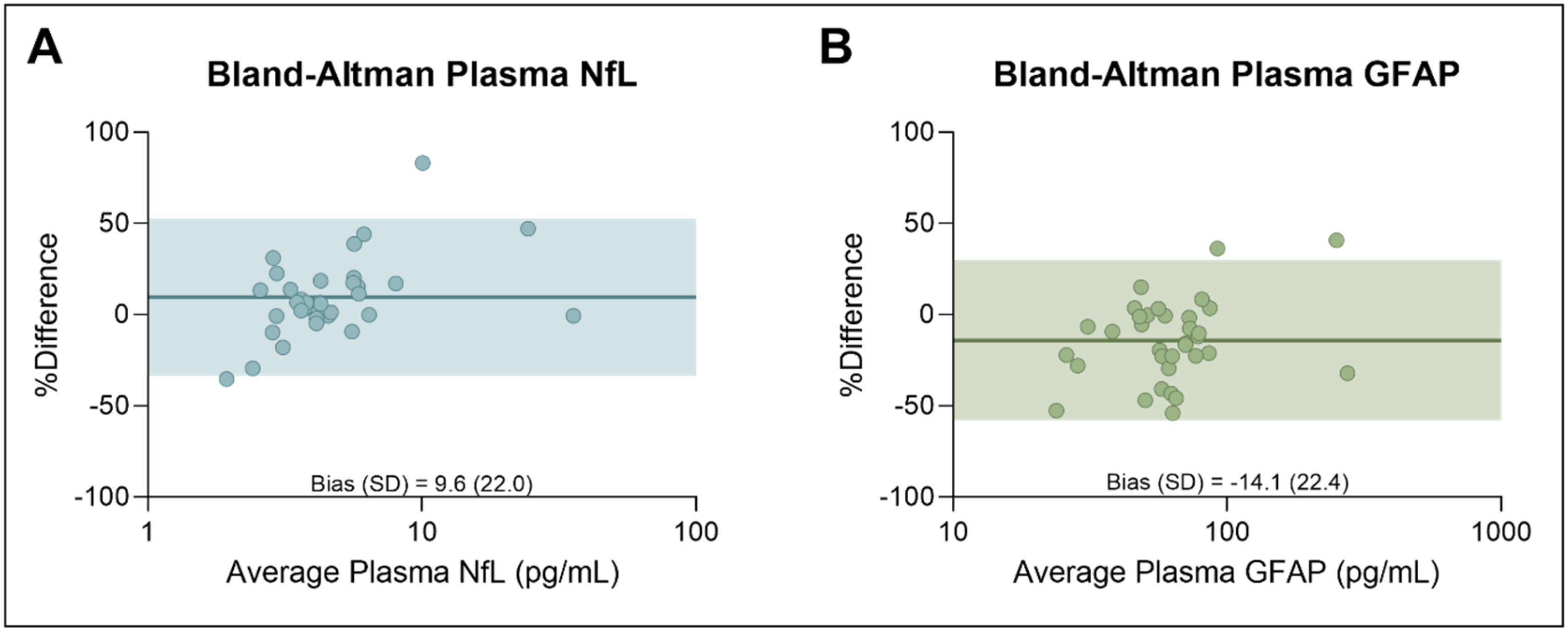

Supplemental Figure 1: Bland-Altman plots of n=34 plasma samples included in cross lot analysis of NfL (A) and GFAP (B) for the SHRed cohort. N=35 samples from the original Neurology-4-plex B (N4PB) lot (503228) were selected to be run on the second N4PB lot (503475), selected to cover the full dynamic range. N=1 outlier was excluded from the final analysis of both analytes using Tukey's test. **A)** The shaded region represents the 95% limits of agreement, and each datapoint represents the difference between NfL levels from the two N4PB lots for an individual sample. Bland Altman plot for NfL data showed bias of 9.6% (SD=22.0; 95% CI = -33.5 – 52.6). **B)** The shaded region represents the 95% limits of agreement, and each datapoint represents the difference between GFAP levels from the two N4PB lots for an individual sample. Bland Altman plot for GFAP data showed bias of -14.1% (SD=22.4; 95% CI = -58.0 – 29.8). **Abbreviations**: N4PB – Neurology 4-plex B, NfL – neurofilament light, GFAP – glial fibrillary acidic protein, SD – standard deviation.

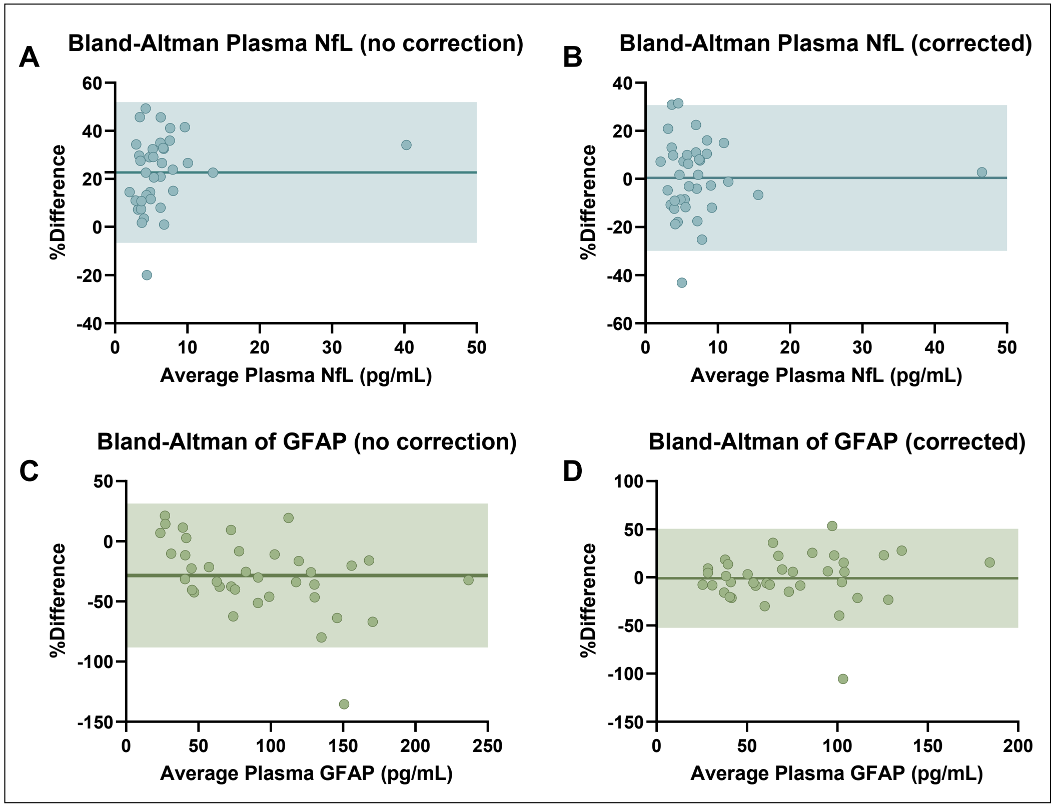

Supplemental Figure 2. Cross assay comparison of NfL and GFAP using the N4PE and N4PB assays. n = 37 specimens from the SHRed cohort were run on Neurology-4-plex B (Lot: 503228/503475) and Neurology-4-plex E (Lot: 503940) and compared using Bland-Altman analysis. Weighted Deming Regression was used to harmonize the N4PB values to N4PE values. The shaded region represents the 95% limits of agreement, and each datapoint shows the difference in an individual's NfL (A/B) and GFAP (C/D) levels as measured by the N4PE and N4PB assays. **A)** Bland Altman plot for uncorrected NfL data showed bias of 22.7% (SD=14.9; 95% CI = -6.5 – 51.9) and a correlation of rho=0.92. **B)** Bland Altman for corrected NfL data showed bias of 0.44 (SD=15.5; 95% CI = -29.8 – 30.7). **C)** Bland Altman plot for uncorrected GFAP data showed bias of -28.4% (SD=30.5; 95% CI = -88.3 – 31.5) and a correlation of rho=0.85. **D)** Bland Altman for corrected GFAP data showed bias of -0.94 (SD=26.2; 95% CI = -52.4 – 50.5). **Abbreviations**: NfL – neurofilament light, GFAP – glial fibrillary acidic protein, SD – standard deviation, 95% CI – 95% confidence interval, N4PE – Neurology-4-Plex E, N4PB – Neurology-4-Plex B.

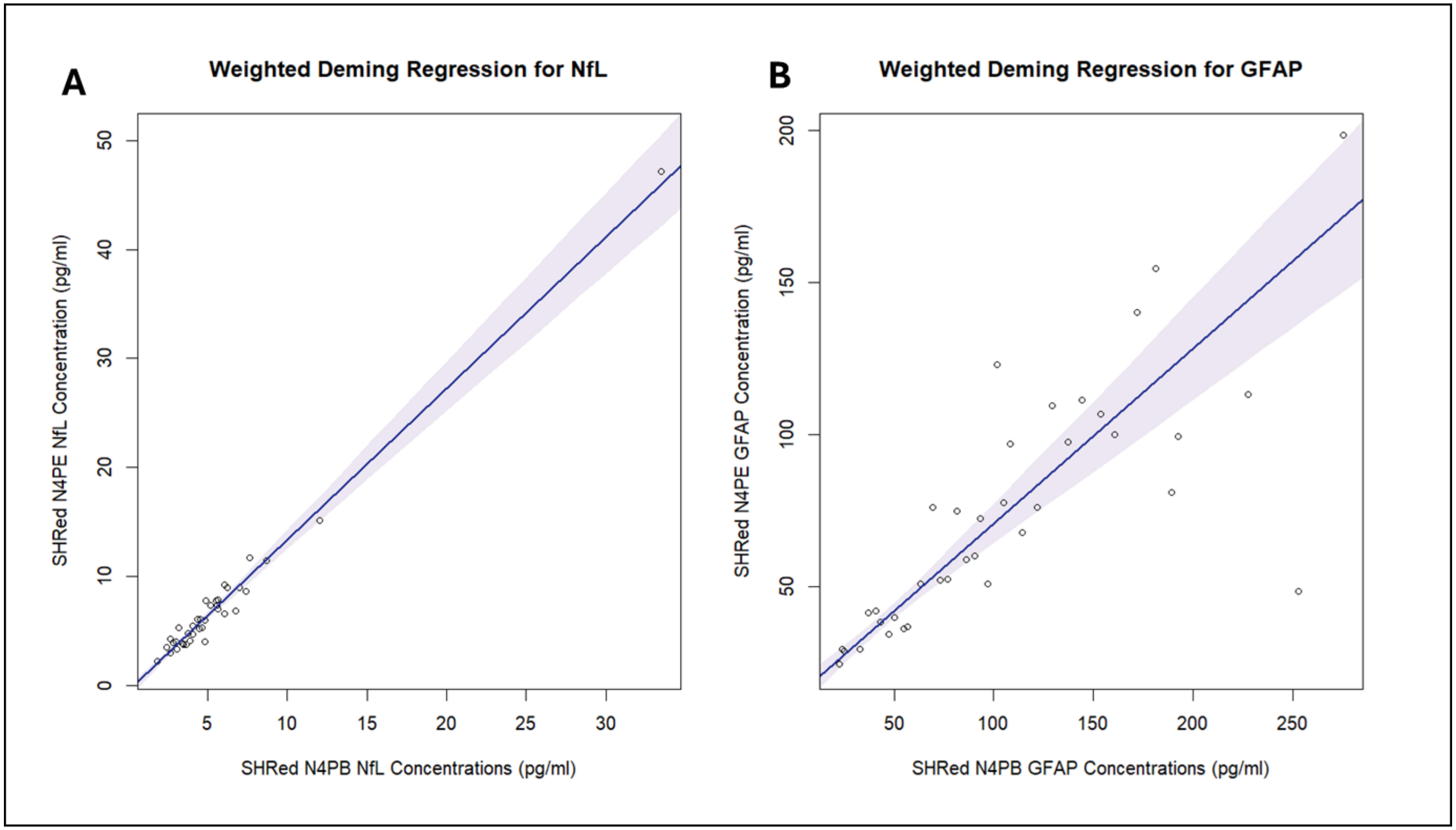

Supplemental Figure 3: **Weighted Deming Regression of cross-assay analysis (n=36 participants) for NfL (A) and GFAP (B). A)** Each point represents an individual's NfL level as measured by the N4PB and N4PE assay. Weighted Deming Regression equation for NfL: N4PE = 1.39*N4PB – 0.59. The confidence interval (CI) for the slope and intercept was (estimate (95% CI)) 1.39 (1.26 – 1.55) and -0.59 (-1.35 – -0.07) respectively. N4PB and N4PE NfL levels had Spearman's correlation of 0.922. **B)** Each point represents an individual's GFAP level as measured by the N4PB and N4PE assay. Weighted Deming Regression equation for GFAP: N4PE = 0.57*N4PB + 13.4. The confidence interval (CI) for the slope and intercept was (estimate (95% CI)) 0.57 (0.47 – 0.68) and 13.40 (8.29 – 18.60) respectively. N4PB and N4PE GFAP levels had Spearman's correlation of 0.855. **Abbreviations**: NfL – neurofilament light, GFAP – glial fibrillary acidic protein, N4PE - Neurology 4-plex E assay, N4PB – Neurology 4-plex B assay.

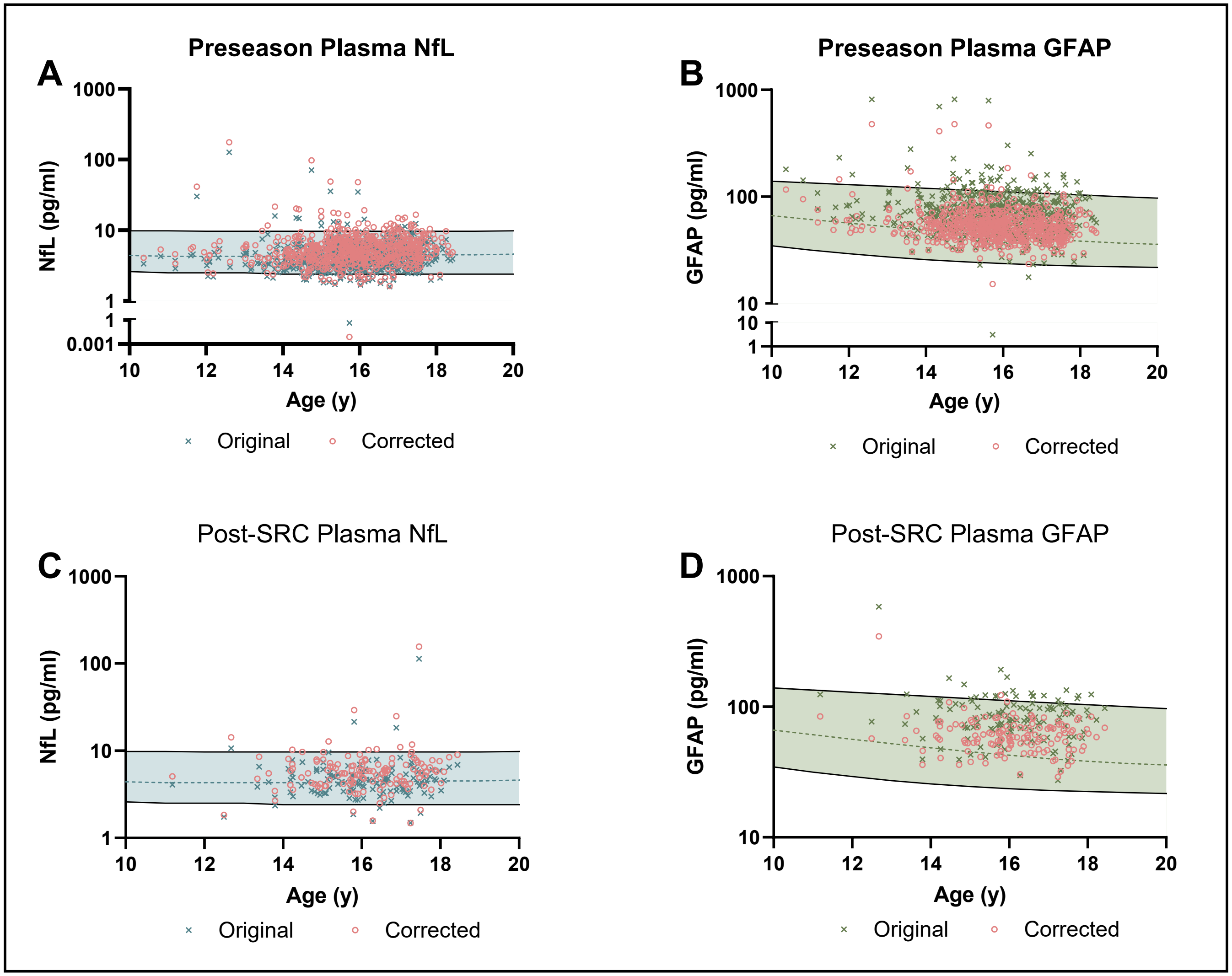

Supplemental Figure 4: Original and corrected SHRed data for NfL and GFAP concentrations at preseason (n=658) and post-SRC (n=134) overlayed over their respective reference intervals. Green Xs represent the original values, and red circles represent corrected values. **Abbreviations**: NfL – neurofilament light; GFAP – glial fibrillary acidic protein, SRC- sport-related concussion.

	Log(NfL pg/mL)		Log(GFAP pg/mL)	
	β	p-value	β	p-value
Timepoint [SRC]	**0.04812**	**0.0483**	**0.05190**	**0.0005**
Age	0.002950	0.8776	**-0.03549**	**0.0024**
Sex [Male]	**0.01514**	**0.0379**	**-0.02136**	**<0.0001**
BMI	**-0.01147**	**<0.0001**	**-0.007252**	**<0.0001**
Days Since SRC	**0.005237**	**0.0323**	**0.004941**	**0.0010**
Collision Sport [Y]	-0.04046	0.0927	**-0.04309**	**0.0034**

Supplemental Table 1: Bivariate regressions of clinical variables with log NfL and GFAP values. Bivariate linear regression model (n=792) incorporated log transformed biomarker data, whether a sample was collected at baseline or post-SRC (timepoint), sex, age, BMI, and days since SRC. Significant associations are bolded. n=107 participants were excluded from analysis due to missing data. **Abbreviations**: SRC – Sports-related concussion, BMI – Body Mass Index, NfL – neurofilament light, GFAP – glial fibrillary acidic protein.
*Bolded = significant p-value

	Log(NfL pg/mL)		Log(GFAP pg/mL)	
	β	p-value	β	p-value
Timepoint [SRC]	0.002534	0.9446	0.02664	0.2282
Age	**0.02538**	**0.0005**	**-0.01759**	**<0.0001**
Sex [Male]	0.02652	0.1595	**-0.02242**	**0.0497**
BMI	**-0.01352**	**<0.0001**	**-0.005839**	**<0.0001**
Days Since SRC	0.005294	0.1501	0.003103	0.1636
Collision Sport [Y]	-0.01363	0.6037	-0.01344	0.3979

Supplemental Table 2. Associations of plasma biomarkers, demographic variables, and preseason or post SRC sampling. Multivariable linear regression model (n=792) incorporated log transformed biomarker data, whether a sample was collected at baseline or post SRC (timepoint), age, sex, BMI, and days since SRC. For categorical variables baseline and female sex were used as reference values. n=107 participants were excluded from analysis due to missing data. **Abbreviations**: NfL – neurofilament light, GFAP – glial fibrillary acidic protein, SRC – sports-related concussion, BMI – body mass index.
*Bolded = significant p-value

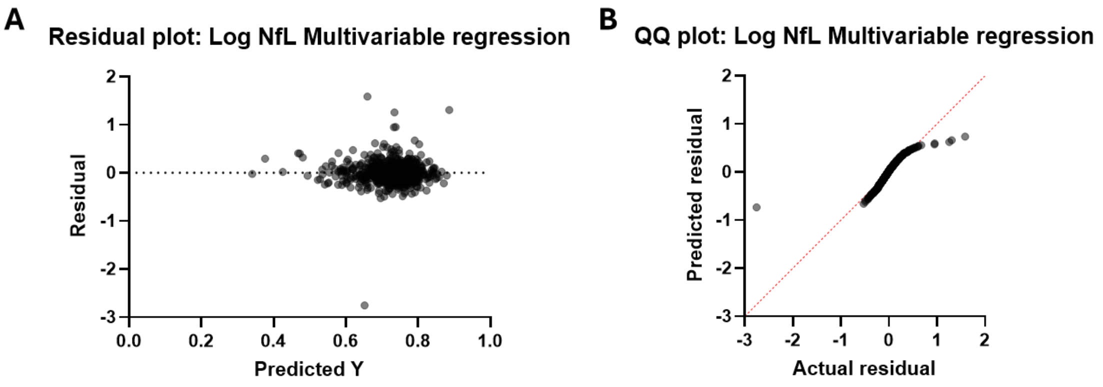

C

Multicollinearity	Variable	VIF
β0	Intercept	
β1	Timepoint[Post-SRC]	2.403
β2	Sex[1]	1.039
β3	Age	1.061
β4	BMI	1.083
β5	Days since SRC	2.417

Supplemental Figure 5: **Illustration of normality of residuals, linearity, and absence of multicollinearity for multivariable regression for log NfL**. **(A)** Residual plot of the multivariable regression analysis for log NfL. **(B)** QQ plot of the multivariable regression analysis for log NfL. **(C)** Multicollinearity table of variables used in the multivariable regression for log NfL. The p-values for the variables used in this multivariable regression can be found in Supplemental Table 2. n=792 participants were available for analysis and n=107 participants were excluded due to missing data. **Abbreviations**: NfL – neurofilament light, SRC – sports-related concussion, BMI – body mass index.

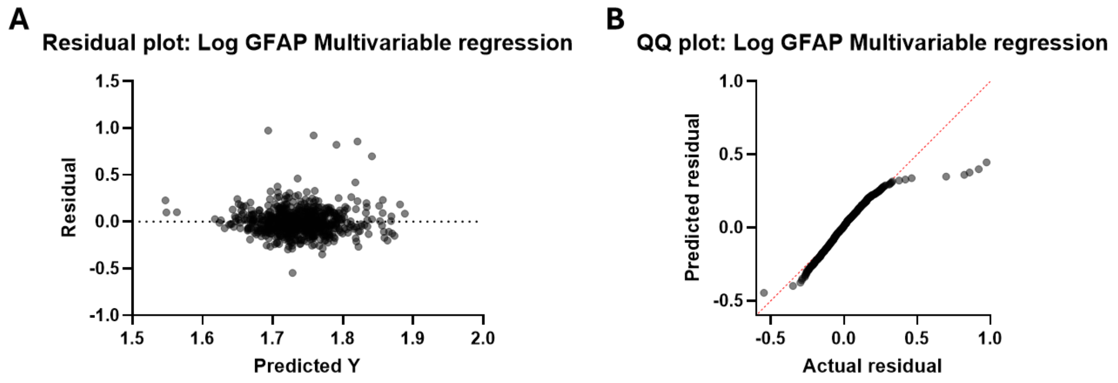

C

Multicollinearity	Variable	VIF
β0	Intercept	
β1	Timepoint[Post-SRC]	2.403
β2	Sex[1]	1.039
β3	Age	1.061
β4	BMI	1.083
β5	Days since SRC	2.417

Supplemental Figure 6: **Illustration of normality of residuals, linearity, and absence of multicollinearity for multivariable regression for log GFAP**. **(A)** Residual plot of the multivariable regression analysis for log GFAP. **(B)** QQ plot of the multivariable regression analysis for log GFAP. **(C)** Multicollinearity table of variables used in the multivariable regression for log GFAP. The p-values for the variables used in this multivariable regression can be found in Supplemental Table 2. n=792 participants were available for analysis and n=107 participants were excluded due to missing data. **Abbreviations**: GFAP – glial fibrillary acidic protein, SRC – sports-related concussion, BMI – body mass index.

NfL	Post-SRC 0-3 days (n=35)				Post-SRC 4-10 days (n=65)				Post-SRC 11-28 days (n=34)			
	Expected	Observed	Percent difference	95% Confidence Interval	Expected	Observed	Percent difference	95% Confidence Interval	Expected	Observed	Percent difference	95% Confidence Interval
**< 5th percentile**	2 (5%)	3 (9%)	57.1	(-226.4 – 351.5)	3 (5%)	1 (2%)	-61.5	(-227.6 – 82.0)	2 (5%)	1 (3%)	-58.8	(-331.8 – 195.7)
**5th – 50th percentile**	16 (45%)	9 (26%)	-44.4	(-90.4 – 5.5)	29 (45%)	17 (26%)	-41.0	(-75.6 – -4.5)*	15 (45%)	6 (18%)	-58.8	(-102.8 – -10.3)*
**51st – 95th percentile**	16 (45%)	22 (63%)	38.1	(-13.7 – 86.0)	29 (45%)	42 (65%)	44.4	(6.4 – 80.0)*	15 (45%)	23 (68%)	52.3	(-0.2 – 99.3)*
**> 95th percentile**	2 (5%)	1 (3%)	-57.1	(-323.4 – 190.8)	3 (5%)	5 (8%)	61.5	(-121.8 – 256.5)	2 (5%)	4 (12%)	117.6	(-182.2 – 434.5)
**< 50th percentile**	18 (50%)	12 (34%)	-34.3	(-77.1 – 12.0)	33 (50%)	18 (28%)	-46.2	(-77.2 – -12.7)*	17 (50%)	7 (21%)	-58.8	(-98.7 – -13.7)*
**5th–95th percentile**	32 (90%)	31 (89%)	-3.2	(-20.9 – 14.2)	59 (90%)	59 (91%)	0	(-12.0 – 12.0)	31 (90%)	29 (85%)	-6.5	(-25.3 – 11.8)
**> 50th percentile**	18 (50%)	23 (66%)	28.6	(-17.5 – 71.8)	33 (50%)	47 (72%)	43.1	(9.7 – 74.2)*	17 (50%)	27 (79%)	58.8	(13.7 – 98.7)*

Supplemental Table 3: Expected and observed frequencies, percent difference and 95% confidence interval (CI) for each section of the NfL reference interval separated by days since injury. Abbreviations: NfL – neurofilament light.

GFAP	Post-SRC 0-3 days				Post-SRC 4-10 days				Post-SRC 11-28 days			
	Expected	Observed	Percent difference	95% Confidence Interval	Expected	Observed	Percent difference	95% Confidence Interval	Expected	Observed	Percent difference	95% Confidence Interval
**< 5th percentile**	2 (5%)	0 (0%)	-117.6	(-381.9 – 91.5)	3 (5%)	0 (0%)	-89.6	(-247.2 – 21.4)	2 (5%)	0 (0%)	-121.2	(-392.1 – 93.8)
**5th – 50th percentile**	15 (45%)	7 (21%)	-52.3	(-97.3 – -2.9)*	30 (45%)	8 (12%)	-73.0	(-103.4 – -40.4)*	15 (45%)	3 (9%)	-80.8	(-121.8 – -35.1)*
**51st – 95th percentile**	15 (45%)	26 (76%)	71.9	(20.6 – 116.1)*	30 (45%)	59 (88%)	96.2	(62.9 – 125.6)*	15 (45%)	29 (87%)	94.3	(45.8 – 134.9)*
**> 95th percentile**	2 (5%)	1 (3%)	-58.8	(-331.8 – 195.7)	3 (5%)	0 (0%)	-89.6	(-247.2 – 21.4)	2 (5%)	1 (3%)	-60.6	(-340.7 – 200.8)
**< 50th percentile**	17 (50%)	7 (21%)	-58.8	(-98.7 – -13.7)*	34 (50%)	8 (12%)	-77.6	(-104.6 – -47.8)*	17 (50%)	3 (9%)	-84.8	(-120.8 – -43.0)*
**5th–95th percentile**	31 (90%)	33 (97%)	6.5	(-8.0 – 22.8)	60 (90%)	67 (100%)	11.6	(5.3 – 22.3)*	30 (90%)	32 (96%)	6.7	(-8.2 – 23.4)
**> 50th percentile**	17 (50%)	27 (79%)	58.8	(13.7 – 98.7)*	34 (50%)	59 (88%)	74.6	(44.9 – 101.7)*	17 (50%)	30 (90%)	78.8	(37.3 – 115.3)*

Supplemental Table 4: Expected and observed frequencies, percent difference and 95% confidence interval (CI) for each section of the GFAP reference interval separated by days since injury. Abbreviations: GFAP – glial fibrillary acidic protein.

NfL	0-3 days v 4-10 days	0-3 days vs 11-28 days	4-10 days vs 11-28 days
**RI Location**	**p-value**	**p-value**	**p-value**
< 5th percentile	0.2392	0.6274	1
5th – 50th percentile	1	0.6028	0.4823
51st – 95th percentile	0.7906	0.8691	0.9372
> 95th percentile	0.5963	0.3358	0.7633
< 50th percentile	0.6473	0.3154	0.5968
5th–95th percentile	1	0.9628	0.6267
> 50th percentile	0.6473	0.3154	0.5968
**GFAP**	**p-value**	**p-value**	**p-value**
< 5th percentile	N/A	N/A	N/A
5th – 50th percentile	0.4630	0.3288	0.8516
51st – 95th percentile	0.2772	0.3693	1
> 95th percentile	0.7519	1	0.7403
< 50th percentile	0.4630	0.3288	0.8516
5th–95th percentile	0.7519	1	0.7403
> 50th percentile	0.4630	0.3288	0.8516

Supplemental Table 5: Comparison of the different location of the reference interval across the different time bins for NfL and GFAP. A proportion test was used to assess if there were significant differences in the proportions of individuals in various sections of the reference interval various time bins post-SRC. **Abbreviations**: NfL – neurofilament light, GFAP – glial fibrillary acidic protein, RI – reference interval.

NfL	5th-95th percentile	> 95th percentile	p-value
Preseason	580	51	>0.9999
Post-SRC	119	10	
**GFAP**	**5th-95th percentile**	**> 95th percentile**	**p-value**
Preseason	644	12	>0.9999
Post-SRC	132	2	

Supplemental Table 6: **Fisher's Exact Test comparing data within RI (5th-95th percentile) and above the RI (>95th percentile) at preseason and post-SRC for NfL and GFAP.** **Abbreviations**: SRC – Sports-related concussion, NfL – neurofilament light, GFAP – Glial Fibrillary Acidic Protein.

			SCAT 5 Section (Range of possible scores)										
	**GFAP**	**NfL**	**No. of Symptoms (0-22)**	**Symptom Severity (0-132)**	**Orientation (0-5)**	**Immediate Memory (0-30)**	**Concentration (0-4)**	**Balance Errors (0-30)**	**Delayed Recall (0-10)**	**Days Since Concussion**	**Days to RTP**	**BMI**	**MSK Injury (Y/N)**
**Person 1**	58.0	10.4	22	103	5	22	5	12	7	4	26	22.4	Y
**Person 2**	81.0	12.8	20	34	4	23	2	14	5	12	77	26.5	Y
**Person 3**	82.0	10.2	14	23	5	22	3	5	8	6	20	22.9	N
**Person 4**	57.6	25.0	21	60	5	26	2	10	10	5	26	19.8	N
**Person 5**	60.7	11.8	15	35	5	24	4	0	9	6	73	23.2	Y
**Person 6**	89.7	156.3	13	36	5	22	3		5	16	0	19.0	Y
**Person 7**	67.6	11.0	7	11	0	N/A	N/A	N/A	N/A	11	29	22.7	N
**Person 8**	71.1	10.3	N/A	N/A	0	N/A	N/A	N/A	N/A	13	34	20.5	N
**Person 9**	346.2	14.3	9	25	5	16	3	12	5	2	13	18.2	N
**Person 10**	56.1	29.2	7	22	0	N/A	N/A	N/A	N/A	10	59	22.6	N
**Person 11**	122.9	6.7	3	4	5	19	3	10	3	22	31	20.7	N

Supplemental Table 7: **Case Report Table of individuals above the 95th percentile for individual post-SRC sample taken within 28 days**. Reporting values for those above the 95th percentile for GFAP and NfL. The shaded boxes represents whether the individual was above the 95th percentile for GFAP and/or NfL. **Abbreviations**: SRC – Sports-related concussion, BMI – Body Mass Index, No. – number, SCAT5 – Sports Concussion Assessment Tool 5, IQR – interquartile range, NfL – neurofilament light, GFAP – Glial Fibrillary Acidic Protein, MSK – Musculoskeletal Injury.

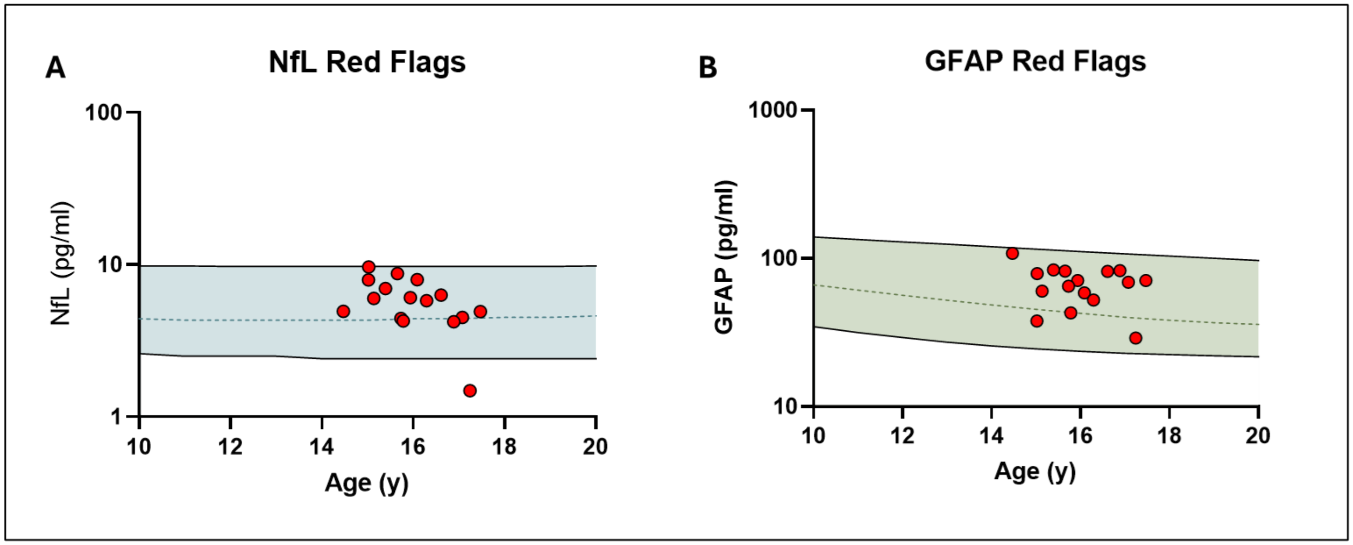

Supplemental Figure 7: Plasma NfL and GFAP concentrations in individuals who were identified with a red flag (n=16) overlayed onto NfL and GFAP reference intervals. A) The shaded region represents the 5th – 95th percentile for NfL with red points representing NfL levels for individuals with red flags. B) The shaded region represents the 5th – 95th percentile for GFAP with red points representing GFAP levels for individuals with red flags. **Abbreviations**: NfL – neurofilament light; GFAP – glial fibrillary acidic protein.

NfL		Preseason			Post-SRC		
		**Lower** (5th-50th percentile)	**Upper** (50th-95th percentile)	**p-value**	**Lower** (5th-50th percentile)	**Upper** (50th-95th percentile)	**p-value**
	**Total n (%)**	209	371		32	87	
No. of Symptoms	**n (%)**	193	338	0.2394	31	80	0.7272
	**Median [IQR]**	5.0 [2.0 – 10.0]	5.0 [1.0 – 9.0]		10.0 [7.0 – 17.0]	12.0 [4.0 – 19.0]	
Symptom Severity	**n (%)**	191	335	0.2612	31	80	0.7251
	**Median [IQR]**	7.0 [2.0 – 16.0]	6.0 [2.0 – 14.0]		21.0 [8.0 – 47.0]	21.0 [7.0 – 42.5]	
Orientation (SCAT5)	**n (%)**	190	333	0.5952	27	68	0.9863
	**Median [IQR]**	5.0 [5.0 – 5.0]	5.0 [4.0 – 5.0]		5.0 [5.0 – 5.0]	5.0 [5.0 – 5.0]	
Immediate Memory (of 30) (SCAT5)	**n (%)**	187	333	0.4479	**26**	**68**	**0.0045**
	**Median [IQR]**	21.0 [19.0 – 23.0]	20.0 [18.0 – 23.0]		**19.0 [17.0 – 20.0]**	**21.0 [19.0 – 24.0]**	
Concentration (SCAT5)	**n (%)**	190	332	0.2902	26	68	0.2027
	**Median [IQR]**	3.0 [3.0 – 4.0]	3.0 [3.0 – 4.0]		3.0 [2.0 – 4.0]	3.0 [3.0 – 4.0]	
Balance Errors (SCAT5)	**n (%)**	189	332	0.9888	26	67	0.7538
	**Median [IQR]**	3.0 [1.0 – 5.0]	3.0 [1.0 – 5.0]		6.0 [4.0 – 9.3]	6.0 [3.0 – 9.0]	
Delayed Recall (SCAT5)	**n (%)**	178	320	0.1225	25	68	0.2375
	**Median [IQR]**	7.0 [6.0 – 8.0]	7.0 [5.0 – 8.0]		6.0 [5.0 – 7.0]	6.0 [5.0 – 8.0]	
Previous Concussion No.	**n (%)**	205	360	0.6781	32	87	0.9163
	**Median [IQR]**	0.0 [0.0 – 1.0]	0.0 [0.0 – 1.0]		1.0 [0.0 – 1.0]	0.0 [0.0 – 1.0]	
BMI	**n (%)**	**184**	**312**	**0.0083**	**28**	**76**	**0.0079**
	**Median [IQR]**	**23.0 [20.6 – 26.4]**	**22.2 [20.3 – 24.5]**		**24.0 [21.2 – 28.0]**	**21.9 [20.1 – 24.4]**	
Sex	**Males, n (%)**	139	253	0.6449	17	46	>0.9999
	**Females, n (%)**	70	117		15	41	
Days Since Concussion	**n (%)**				32	87	0.5306
	**Median [IQR]**				5.5 [3.0 – 9.8]	6.0 [3.0 – 11.0]	
Days to RTP	**n (%)**				32	87	0.6172
	**Median [IQR]**				21.0 [15.3 – 37.0]	22.0 [15.0 – 42.0]	

c

Supplemental Table 8: Comparison of the 5th – 50th percentile and 50th – 95th percentile for NfL collected within 28 days after injury for post-SRC cohort. A Mann-Whitney U Test was used to analyze continuous variables and Fisher's Exact test was used to analyze categorical variables, between the 5th – 50th percentile and 50th – 95th percentile of the reference interval for NfL.
Abbreviations: SRC – Sports-related concussion, BMI – Body Mass Index, No. – number, SCAT5 – Sports Concussion Assessment Tool 5, IQR – interquartile range, NfL – neurofilament light.
*Bolded = significant p-value

GFAP		Preseason Lower (5th-50th percentile)	Preseason Upper (50th-95th percentile)	Preseason p-value	Post-SRC Lower (5th-50th percentile)	Post-SRC Upper (50th-95th percentile)	Post-SRC p-value
	**Total n (%)**	181	463		18	114	
No. of Symptoms	**n (%)**	**153**	**440**	**0.0066**	17	105	0.6742
	**Median [IQR]**	**4.0 [1.0 – 8.5]**	**5.0 [2.0 – 10.0]**		11.0 [6.0 – 16.5]	12.0 [6.5 – 18.5]	
Symptom Severity	**n (%)**	**149**	**438**	**0.0205**	17	105	0.8532
	**Median [IQR]**	**5.0 [1.0 – 12.0]**	**7.0 [2.0 – 16.0]**		27.0 [6.5 – 36.5]	22.0 [8.5 – 44.0]	
Orientation (SCAT5)	**n (%)**	150	433	0.3377	14	90	0.4792
	**Median [IQR]**	5.0 [5.0 – 5.0]	5.0 [4.0 – 5.0]		5.0 [5.0 – 5.0]	5.0 [4.8 – 5.0]	
Immediate Memory (of 30) (SCAT5)	**n (%)**	148	432	0.7477	**14**	**89**	**0.0198**
	**Median [IQR]**	21.0 [19.0 – 22.0]	20.0 [18.0 – 23.0]		**18.5 [16.8 – 20.0]**	**21.0 [19.0 – 23.0]**	
Concentration (SCAT5)	**n (%)**	150	432	0.3575	14	89	0.1492
	**Median [IQR]**	3.0 [3.0 – 4.0]	3.0 [3.0 – 4.0]		3.0 [2.0 – 3.3]	3.0 [3.0 – 4.0]	
Balance Errors (SCAT5)	**n (%)**	150	430	0.4983	13	88	0.8663
	**Median [IQR]**	3.0 [1.0 – 5.0]	3.0 [1.0 – 5.0]		6.0 [3.0 – 8.0]	6.0 [3.0 – 9.0]	
Delayed Recall (SCAT5)	**n (%)**	**141**	**471**	**0.0340**	13	89	0.3616
	**Median [IQR]**	**7.0 [6.0 – 8.0]**	**7.0 [5.0 – 8.0]**		6.0 [4.5 – 7.5]	60. [5.0 – 8.0]	
Previous Concussion No.	**n (%)**	171	458	0.8109	18	114	0.4986
	**Median [IQR]**	0.0 [0.0 – 1.0]	0.0 [0.0 – 1.0]		0.0 [0.0 – 1.0]	1.0 [0.0 – 1.0]	
BMI	**n (%)**	**146**	**411**	**0.0219**	**17**	**99**	**0.0025**
	**Median [IQR]**	**23.1 [20.9 – 26.6]**	**22.3 [20.5 – 24.9]**		**25.0 [22.4 – 29.4]**	**22.3 [20.1 – 24.4]**	
Sex	**Males, n (%)**	130	303	0.1356	**14**	**57**	**0.0403**
	**Females, n (%)**	51	159		**4**	**57**	
Days Since Concussion	**n (%)**				18	114	0.5424
	**Median [IQR]**				5.5 [3.0 – 9.3]	6.0 [4.0 – 11.0]	
Days to RTP	**n (%)**				18	114	0.5568
	**Median [IQR]**				20.5 [13.0 – 43.5]	22.0 [15.0 – 41.3]	

Supplemental Table 9: Comparison of the 5th – 50th percentile and 50th – 95th percentile for GFAP collected within 28 days after injury for post-SRC cohort. A Mann-Whitney U Test was used to analyze continuous variables and a Fisher's Exact Test was used to analyze categorical variables, between the 5th – 50th percentile and 50th – 95th percentile of the reference interval for GFAP. **Abbreviations**: SRC – Sports-related concussion, BMI – Body Mass Index, No. – number, SCAT5 – Sports Concussion Assessment Tool 5, IQR – interquartile range, GFAP – glial fibrillary acidic protein.
*Bolded = significant p-value

	**Post-SRC <95th percentile**	**Post-SRC Red Flags**	**Post-SRC >95th percentile**
**Median [IQR]**	21.0 [15.0 – 41.0]	44.0 [20.3 – 56.3]	29.0 [14.8 – 52.8]

Supplemental Table 10: **Median RTP for different post-SRC sub-cohorts.** n=123 post-SRC <95th percentile. n=16 post-SRC Red Flags. n=11 post-SRC >95th percentile. **Abbreviations**: RTP – Return-to-play, IQR – interquartile range.

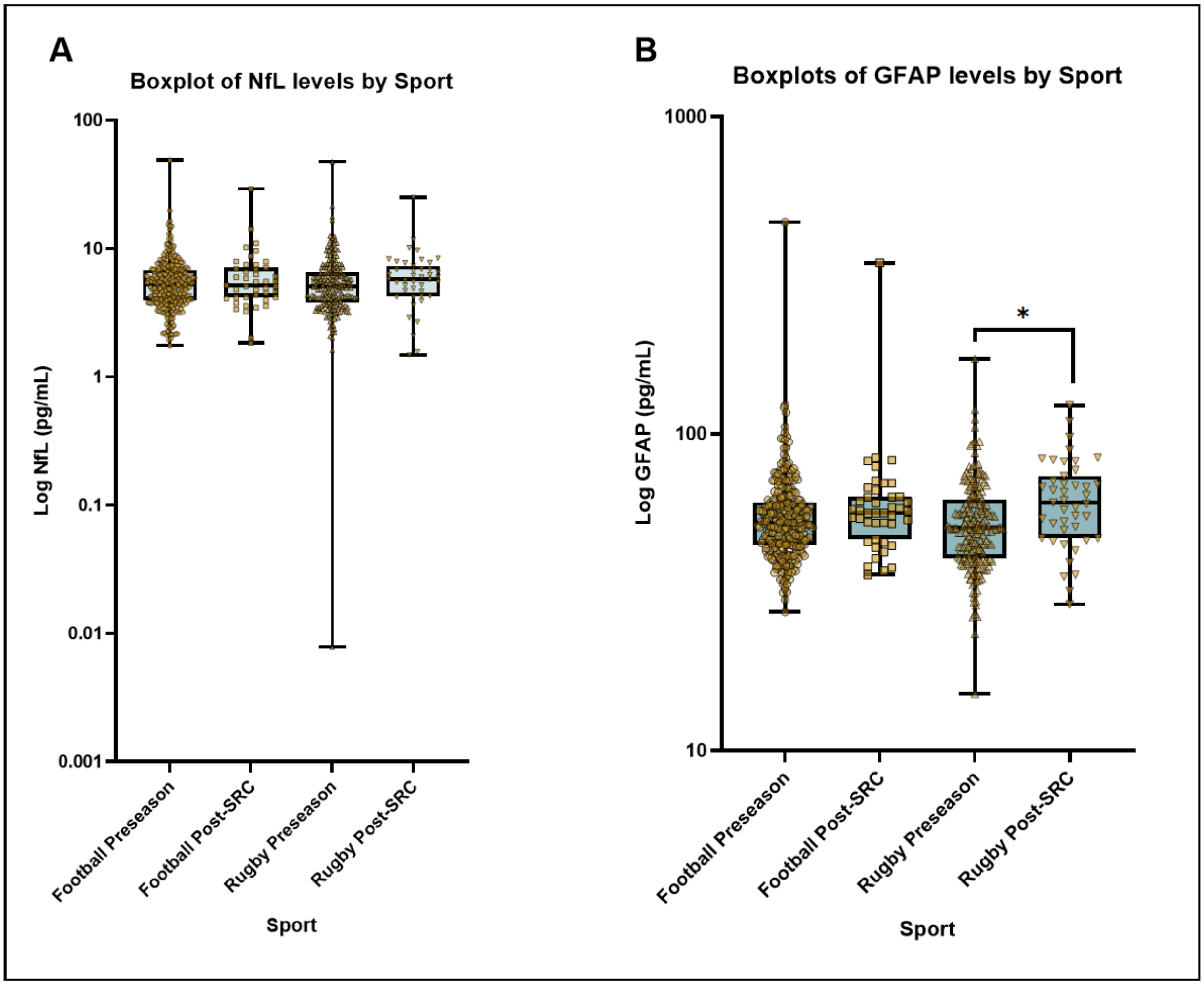

Supplemental Figure 8: **Boxplot of A) NfL levels and B) GFAP levels at preseason and post-SRC for football (preseason/post-SRC; n=295/n=43) and rugby (preseason/post-SRC; n=199/n=43).** Only sports that had more than 10 athletes participating were selected. Each point represents an individual's NfL (A) and GFAP (B) level stratified by sport and timepoint. A Mann-Whitney U test was used to compare biomarker levels within each sport. GFAP levels for rugby were significantly different between preseason and post-SRC (p=0.0015; Mann-Whitney U = 2968). **Abbreviations**: NfL – neurofilament light, GFAP – glial fibrillary acidic protein.

**Post-SRC group**			
	**1-3 days**	**4-10 days**	**11-28 days**
**N (%)**	35 (26%)	65 (49%)	34 (25%)
**Days Since SRC (median, IQR)**	2 (2 – 3)	6 (5 – 8)	16 (12 – 19.5)
**Days to Return-to-Play (RTP) (median, IQR)**	16 (13 – 37)	22 (17 – 40.5)	33 (16.8 – 52.5)
**Male Sex (N, %) ***	18 (51%)	38 (58%)	16 (47%)
**Female Sex (N, %) ***	17 (49%)	27 (42%)	18 (53%)

Supplemental Table 11: **Breakdown of various datapoints for the post-SRC time bins**. **Abbreviations**: SRC – sports-related concussion, RTP – Return-to-play, IQR – interquartile range.
